# Polymeric Infrared and Fluorescent Probes to Assess Macrophage Diversity in Bronchoalveolar Lavage Fluid of Asthma and Other Pulmonary Disease Patients

**DOI:** 10.3390/polym16233427

**Published:** 2024-12-05

**Authors:** Igor D. Zlotnikov, Elena V. Kudryashova

**Affiliations:** Faculty of Chemistry, Lomonosov Moscow State University, Leninskie Gory, 1/3, 119991 Moscow, Russia; zlotnikovid@my.msu.ru

**Keywords:** bronchoalveolar lavage fluid, IR marker, CD206, macrophage, diagnosis

## Abstract

Bronchial asthma remains a serious medical problem, as approximately 10% of patients fail to achieve adequate symptom control with available treatment options. Macrophages play a pivotal role in the pathophysiology of asthma, as well as in some other respiratory disorders. Typically, they are classified into two major classes, M1 and M2; however, recent findings have indicated that in fact there is a whole range of macrophage polarization and functional diversity beyond this bimodal division. The isolation of individual cell sub-populations and the identification of their role and diagnostic/therapeutic significance is still a challenge. Here, we have attempted to assess the differences between patient-derived macrophage populations from bronchoalveolar lavage fluid (BALF) samples in different pulmonary disease conditions, based on their capability to interact with a range of specific and relatively non-specific carbohydrate-based ligands (containing galactose (linear or cyclic form), mannose, trimannose, etc.). Obviously, the main target of these ligands was CD206; however, other minor receptors, able to bind carbohydrates, have also been reported for macrophages. Trimannose binds most specifically to CD206 macrophage receptors, while monomannose has intermediate affinity, and galactose has low affinity and may involve binding to other receptors. This clearly indicates the ligands were chosen based on their predicted binding strength and specificity for CD206, providing the rationale for the study. In some cases, the activated macrophage affinity to galactose base ligands was higher than that to mannose, indicating that complexes of CD206 or other carbohydrate-binding receptors may contribute substantially to macrophage functional features. In addition, variations in receptor clustering and distribution may substantially affect affinity to the same ligand. Interestingly, with a panel of 6–10 different carbohydrate-based ligands with FTIR or fluorescent marker, we were able not only to distinguish between healthy and disease states but also between closely related diseases such as purulent endobronchitis, obstructive bronchitis, pneumonia, and bronchial asthma. For further investigation, specific sub-populations of macrophages, seen as hallmarks to specific diseases, can be isolated and studied separately, likely giving new insights with diagnostic and therapeutic significance for hard-to-treat patients. The group of patients with resistant disease can also be identified with this approach as a fingerprint method to find a more targeted therapeutic strategy, improving their clinical outcomes. As expected, this will provide a large additional array of data for analysis, compared to the work going on in the world. The dataset used by other researchers mainly for known “antibody” ligands is semi-quantitative and insufficient for the purposes of typing as yet unknown and uncomplicated sub-populations. The analysis of the presented data in combination with personalized information from patients’ medical records will be carried out using both traditional methods and machine learning methods.

## 1. Introduction

Respiratory tract disorders such as asthma constitute a group of persistent inflammatory conditions characterized by airway obstruction of the respiratory tract, heightened bronchial responsiveness, and recurrent episodes of dyspnea, coughing, wheezing, and chest tightness [1,2,3,4,5]. This group encompasses bronchial asthma, chronic obstructive pulmonary disease (COPD), and other related conditions.

The underlying causes of these illnesses remain incompletely understood, although it is believed that they may be linked to genetic predisposition, allergen exposure, viral and bacterial infections, tobacco smoke, and other irritants. Factors that increase the risk include having family members with asthma or other allergies, smoking, environmental pollution, and specific occupational exposures [6,7].

Asthma is a persistent inflammatory condition of the respiratory system, characterized by a wide range of symptoms, such as wheezing, coughing, dyspnea, and chest tightness [7,8,9,10,11,12]. At present, there are a variety of therapeutic options available, including inhaled corticosteroids, β-agonists, leukotriene inhibitors, and theophylline, among others. Nevertheless, approximately 10% of individuals with severe asthma fail to achieve satisfactory symptom control, necessitating the exploration of novel strategies and medications.

Macrophages, immune cells responsible for eliminating bacteria and producing biologically active substances that regulate inflammatory responses, play a critical role in the pathogenesis of inflammation in asthma and other diseases of respiratory diseases [8,13,14]. These cells can be classified into two main types: M1 macrophages and M2 macrophages. M2 macrophages, in turn, can be divided into subtypes M2a, M2b, M2c, etc. [9,15,16].

Each patient’s macrophage profile has a unique “fingerprint”, their specific phenotype, which is not yet fully understood. However, analysis of this fingerprint could provide valuable insights into the polarization and activation status of macrophages, allowing for more accurate diagnosis of respiratory tract diseases and potentially targeting key points in the immune system for intervention.

This paper presents a novel approach to the diagnosis of respiratory tract disorders based on a multifaceted analysis of bronchoalveolar lavage fluid (BALF). This concept revolves around the examination and categorization of macrophages within BALF that exhibit the expression of mannose receptors, specifically CD206 [16,17,18,19,20,21]. This methodology can be further developed to encompass the analysis of additional macrophage receptors, thereby enhancing the accuracy of diagnosis and facilitating the formulation of effective treatment strategies for respiratory illnesses. BAL is a procedure involving the aspiration of fluid from the bronchi and alveoli of the lungs, making it a diagnostic tool that provides valuable insights into respiratory health [7,22,23,24,25,26]. A small volume of sterile saline solution is introduced into the airway, followed by its extraction for analysis. The process is meticulously monitored under the guidance of a bronchoscope, which allows the doctor to visually monitor the process.

In the realm of medical diagnostics, BAL serves several critical purposes [27,28,29,30,31,32,33]:

1. Detection of infectious agents.
2. Assessment of immunological disorders.
3. Diagnosis of lung tumors.
4. Identification of idiopathic pulmonary fibrosis.
5. Assessment of treatment efficacy. BAL can be employed to evaluate the effectiveness of treatments for lung conditions such as asthma and chronic obstructive pulmonary disease (COPD).

BALF is particularly useful in the diagnosis of interstitial lung diseases, which affect the interstitial tissue of the lungs. By examining the BALF samples, characteristic alterations in cell populations and protein–receptor profiles can be identified, providing valuable insights into the nature of these conditions. Through an analysis of the BALF samples, the specific causative agents of infection can be determined, along with their susceptibility to antibiotics. Additionally, the BALF technique can be employed to diagnose other pulmonary conditions such as pneumonia, tuberculosis, and pulmonary emphysema.

Recently, researchers explored an approach to the detection and monitoring of respiratory disorders based mainly on metabolomic analysis using gas chromatography–mass spectrometry (GC-MS), where metabolic changes in various lung diseases, including acute pulmonary toxicity, inflammation, asthma, bronchitis, and fibrosis were studied [34]. The application of NMR spectroscopy in the investigation of BALF is also a subject of active research [35,36,37]. Current methods, such as flow cytometry [38,39,40] and immunohistochemistry [41,42], rely on a limited number of biomarkers (mainly antibody-based) and cannot distinguish between subtle phenotypic differences between macrophage types that are individual for each patient. For example, the researchers examined 36 cases of verified lung cancer [43] in order to characterize the disease state. The macrophage markers CD206, CD163, CD80, CD86, CD40, CD45, and CD68 were evaluated using flow cytometry. However, the analysis revealed only subtle variations in these markers. This showed the limits in the ability to make accurate predictions for individual patients with such a method. In addition, a reliable method for determining white blood cells in the BALF using flow cytometry has been developed [44]. However, this method also does not allow one to establish relationships with the clinical picture of the disease.

In this study, a novel approach to diagnosing respiratory tract diseases based on specific carbohydrate-based ligands is suggested to quantify macrophage subpopulations (M1, M2, and their subtypes) in patients with the potential to enhance treatment efficiency. This methodology is predicated on a comprehensive analysis of BALF, encompassing the examination and categorization of macrophage subpopulations based on the expression levels of analytically valuable receptors, such as the CD206 mannose receptor of macrophage [10,14,19,45], as well as the CD68 [7,46,47], CD163 [6,8,16,48], and CD301 receptors [10,47]. The research is aimed at developing a set of carbohydrate-based ligands as specific infrared and fluorescent markers for fingerprint analysis of macrophage subpopulations in BALF for subsequent diagnostics. It is expected that the proposed approach makes it possible to quantify macrophage subpopulations (M1, M2, and their subtypes) in patients. These “new” specific carbohydrate-based ligands will thus provide about 50 additional “coordinates” according to which BALF typing can be performed. As expected, this will provide a large additional array of data for analysis, compared to the work going on in the world. The dataset used by other researchers only for known “antibody” ligands is semi-quantitative and insufficient for the purposes of typing as yet unknown and uncomplicated sub-populations. The analysis of these data in combination with personalized information from patients’ medical records will be carried out using both traditional methods and machine learning methods.

The advantages of the approach suggested are as follows: (i) quantitative macrophage differentiation into subpopulations: unlike flow cytometry, these probes allow simultaneous detection and differentiation of macrophages based on unique surface markers and intracellular components, surpassing single marker assays; (ii) improved sensitivity due to the combination of several markers: the spectral properties of IR and fluorescent signals make them highly sensitive and more specific than traditional antibody-based methods providing semi-quantitative information; and (iii) potential in medical applications: the polymer nature of these probes allows us to study macrophage dynamics during disease progression and response to treatment.

The spectral approach to macrophage fingerprint analysis developed in this study has the potential to detect respiratory diseases at the early stages, facilitate more accurate diagnoses, and track the efficacy of treatment interventions.

## 2. Materials and Methods

### 2.1. Reagents

Polymers. Polyethyleneimine 1.8 kDa (PEI) was purchased from Sigma Aldrich (St. Louis, MO, USA). Activated PEG 5 kDa (N-succinimidyl ester of mono-methoxy poly(ethylene glycol)) was purchased from Shanghai Macklin Biochemical Technology Co., Ltd (Shanghai, China).

Carbohydrates. Mannan, α-D-mannose (Man), methyl α-D-mannoside (Me-Man), D-galactose, and D-lactose were obtained from Sigma Aldrich (St. Louis, MO, USA). Mannotriose-di-(N-acetyl-D-glucosamine) (triMan-GlcNAc_2_) was obtained from Dayang Chem Co., Ltd. (Hangzhou, China).

Fluorophores. Fluorescein isothiocyanate (FITC) was purchased from Sigma Aldrich (St. Louis, MO, USA).

Chemicals. Carbonyldiimidazole (CDI) was obtained from GL Biochem Ltd. (Shanghai, China) via an intermediary Himprocess (Moscow, Russia). DMSO, NaBH_3_CN, NaOH, salts, acids, and solvents were from Reakhim Production (Moscow, Russia).

All reagents were not less than chemically pure grade.

### 2.2. The Synthesis of IR Markers Based on PEG and Three Types of Carbohydrate Fragments

**Figure 1:**
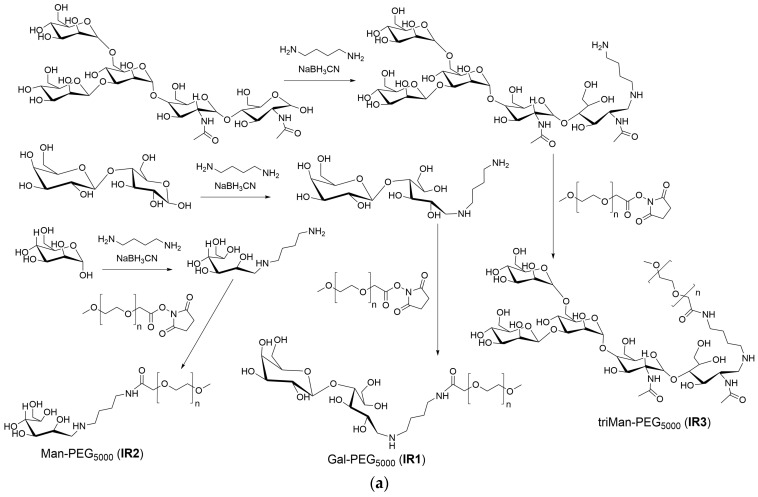

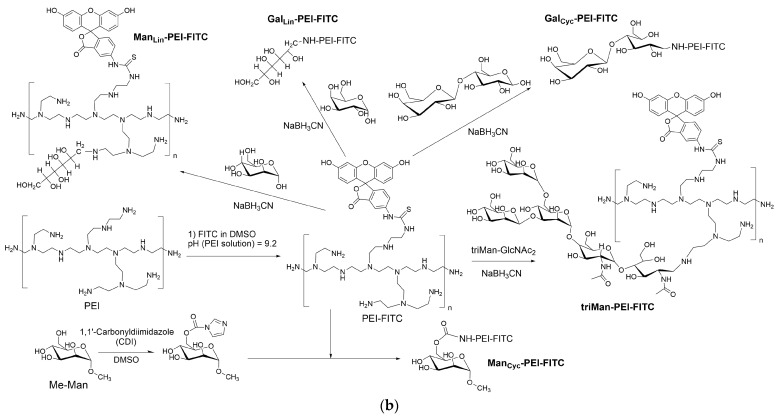
(**a**) A schematic representation of the process of synthesizing IR markers based on polyethylene glycol (PEG) and three distinct types of carbohydrate moieties with varying affinities for CD206 receptors on macrophages, aimed at their identification. (**b**) Another schematic illustration depicting the synthesis of fluorescence-tagged FITC markers incorporating five types of carbohydrates with specific affinities towards CD206 macrophages, designed for their detection.

The synthesis scheme is presented in Figure 1a. The samples of carbohydrates, namely mannose, galactose, and triMan-GlcNAc_2_, weighing 10 mg each, were dissolved in 5 mL of 0.1 mM hydrochloric acid solution.

A mixture of linker putrescine linker and NaBH_3_CN in water, with a 20 percent molar excess of both substances relative to the reducing ends of the carbohydrates, was then added to each of the three samples. The mixtures were incubated at 50 °C for 12 h. Purification of carbohydrate-putrescine samples was performed using dialysis against water (2 h, cut-off 0.5 kDa).

The samples were subjected to freeze-drying, followed by dissolution in a phosphate buffer solution with a pH of 7.4 and a molarity of 10 mM, resulting in a final concentration of 10 mg/mL in carbohydrates. Subsequently, an equimolar equivalent of activated polyethylene glycol 5 kDa (PEG) was added to the samples, gradually and drop-by-drop, while vigorously stirring. The mixture was then incubated at a temperature of 40 °C for a duration of 6 h.

To purify the samples from low-molecular-weight compounds, dialysis against water was employed, conducted twice for 12 h each, with a cut-off of 6–8 kDa. Finally, the samples were freeze-dried to complete the process.

The number of amino groups per putrescine molecule was determined initially and after modifications by TNBS titration (2,4,6-Trinitrobenzenesulfonic acid, final 10 mM). Samples of putrescine or modified forms with TNBS were incubated and kinetic curves A420 were recorded, A420 was determined after an hour and the content of amino groups in the sample was determined (Figure S1) using a molar absorption coefficient of the complex 13,000 M^−1^ × cm^−1^. Sodium borate buffer (50 mM, pH 9.2). T = 22 °C.

### 2.3. The Synthesis of Fluorescent FITC-Labeled Markers Incorporating Five Distinct Types of Carbohydrates

The synthesis scheme is presented in Figure 1b, which provides a comprehensive overview of the process.

Polyethyleneimine 1.8 kDa (PEI) was dissolved in 0.01 M HCl to a concentration of 50 mg/mL. The pH of the solution was then brought to 9.2 by adding a solution of sodium hydroxide and borate buffer. The final concentration of PEI in the solution was 10 mg/mL.

A solution of fluorescein isothiocyanate (FITC) in DMSO at a concentration of 10 mg/mL was slowly added dropwise to the PEI solution until the same molar amount of PEI and FITC was achieved. Purification of PEI-FITC was performed using dialysis against water (6 h, cut-off 3.5 kDa).

The purified PEI-FITC was then divided into five portions. Mannose, galactose, lactose, and triMan-GlcNAc_2_ were added to four portions in a 20-fold molar excess relative to the number of polymer chains in PEI. Sodium borohydride (NaBH_3_CN) was added to 4 samples in a 10 percent molar excess relative to the reducing ends of the carbon backbone. The pH of all reaction mixtures was adjusted to 5 and the samples were incubated at 50 °C for 12 h.

In the case of methyl-α-mannoside (Me-Man), the initial activation of the 6-OH group of the saccharide involved the addition of a solution of carbonyldiimidazole in DMSO to the Me-Man solution in PBS, with a 1.5-fold molar excess of the former. The mixture was incubated at 50 °C for two hours. Subsequently, this solution was slowly added to the PEI-FITC solution, with continuous mixing. The pH was adjusted to 7.4 and the sample was incubated at 50 °C for 6 h.

The purification of all preparations involved a two-step dialysis against water, with a cut-off of 3.5 kDa, for a total of 24 h. All samples were freeze-dried for two days at –60 °C (Edwards 5, BOC Edwards, Sussex, UK).

The additional purification of the samples was accomplished through the use of high-performance liquid chromatography (HPLC), employing gel filtration on the Knauer chromatography system, manufactured by Knauer in Berlin, Germany. A Diasfer-110-C18 column from BioChemMack in Moscow, Russia, with a particle size of 6 µm and dimensions of 4 × 150 mm, was employed. The eluant was a mixture of CH_3_CN and H_2_O in a 90:10 volume ratio. The elution rate was set to 0.8 milliliters per minute, and the temperature was maintained at 25 °C.

Due to the diverse range of conjugate sizes, fractions corresponding to a retention time deviation of ±0.5 min were chosen for the experiments. The degree of PEI modification was assessed through spectrophotometric titration of amino groups before and after the modification procedure using 2,4,6-trinitrobenzene sulfonic acid, a method primarily designed for detecting primary amino groups.

### 2.4. Characterization of Synthesized FTIR and Fluorescent Markers

#### 2.4.1. FTIR Spectroscopy

FTIR spectra of polymer solutions were recorded using a Bruker Tensor 27 spectrometer (Bruker, Bremen, Germany) equipped with a liquid nitrogen-cooled MCT (mercury cadmium telluride) detector. FTIR spectra of solid polymers were recorded using a MICRAN-3 FTIR microscope (Simex, Novosibirsk, Russia) equipped with a liquid nitrogen-cooled MCT detector. For each spectrum, 50–70 scans were accumulated at a 20 kHz scanning speed and averaged. Spectral data were analyzed using the Bruker software program Opus 8.2.28 (Bruker, Bremen, Germany).

#### 2.4.2. NMR Spectroscopy

First, 10–15 mg of the polymers was dissolved in 1 mL of D_2_O. ^1^H NMR spectra of the solutions were recorded on a Bruker DRX-500 instrument, operating at frequencies of 500.13 MHz for ^1^H and 125.76 MHz for ^13^C. The chemical shifts were reported in parts per million (ppm) and were assigned to the corresponding peaks in the NMR spectra of the solvents.

#### 2.4.3. Dynamic Light Scattering (DLS)

The polymer particle size and ζ-potentials were measured using a Zetasizer Nano S «Malvern» (Worcestershire, UK) (4 mW He–Ne-laser, 633 nm, scattering angle 173°) at 25 °C. Experimental data were analyzed using «Zetasizer Software» (v. 8.02).

#### 2.4.4. Determination of Ligand Affinity to Model Mannose Receptor Concanavalin A

The process of complex formation between concanavalin A and FTIR and fluorescent markers was conducted in a PBS buffer, with a pH of 7.4, containing 0.15 M NaCl, 1 mM CaCl_2_, and 1 mM MnCl_2_. The concentration of concanavalin A ranged from 1 to 10 mg/mL, while the concentration of ligands varied from 0.01 to 100 times the estimated dissociation constant (*K*_d_). The reaction took place at 37 °C for 30 min, with continuous stirring. Following the reaction, infrared spectroscopy was employed to record the FTIR spectra of both the protein and the formed complexes, allowing for the determination of dissociation constants using a standardized protocol [21].

### 2.5. Bronchoalveolar Lavage Fluid (BALF) Origin

The BALFs were collected from patients with various diseases of the respiratory tract who were admitted to the Morozov Hospital. The procedure for isolating the BALF followed the methodology outlined in [49]. N-acetylcysteine (ACC) was used to facilitate the dilution of mucus. Cells were extracted after subsequent washing with PBS at a force of 4000× *g* for a duration of 5 min. After the cell isolation procedure, they were comprehensively studied.

### 2.6. Typing Macrophages from BALF and Diagnosis of Diseases

#### 2.6.1. Real-Time FTIR Experiments

The volume of the reaction mixture was 35 µL. The number of cells from BALF was about 2 × 10^5^. The concentration of the IR marker was 5 mg/mL by PEG (equivalent to 1 mM). PBS (0.01 M, pH 7.4). T = 37 °C. The FTIR spectra of BALF samples during real-time (40–60 min) incubation with IR markers of different affinity to CD206 receptors were recorded on the Bruker Tensor 27 spectrometer (Bruker, Bremen, Germany) equipped with a liquid nitrogen-cooled MCT (mercury cadmium telluride) detector. Then, kinetic curves of the intensity changes in the FTIR peaks were analyzed.

#### 2.6.2. «Before and After» FTIR Experiment Technique

The volume of the reaction mixture was 300 µL. The number of cells from BALF was about 5 × 10^5^. The concentration of the IR marker was 2 mg/mL by PEG (equivalent to 0.4 mM), mannan 2 mg/mL. PBS (0.01 M, pH 7.4). T = 37 °C. BALF samples were incubated with IR markers and mannan for 2 h with subsequent washing of the unbound polymers. FTIR spectra were recorded using the MICRAN-3 FTIR microscope (Simex, Novosibirsk, Russia) equipped with a liquid nitrogen-cooled MCT detector.

#### 2.6.3. FTIR Microscopy for Mapping of BALF Samples

BALF samples were incubated for 1 h with triMan-PEG_5000_ (10 mg/mL). Then, half of the samples were treated with mannan (10 mg/mL) for 1 h—as a CD206 high-affinity polymer to exclude non-specific interactions. PBS (0.01 M, pH 7.4). T = 37 °C. FTIR microscopic maps of the peak intensities’ integral distribution in the IR spectra of BALF samples were recorded using the MICRAN-3 FTIR microscope (Simex, Novosibirsk, Russia) equipped with a liquid nitrogen-cooled MCT detector.

#### 2.6.4. Fluorescent Fingerprint Analysis of BALF Samples

The experimental procedure involved the isolation of BALF cells, followed by the application of a cell suspension to the wells of a microtiter plate, where the cells attached. Fluorescent markers were then added, and the mixture was incubated for a period of four hours. The fluorescence of the resulting solution was then analyzed, and binding parameters were calculated. By utilizing five distinct fluorescent markers in both the presence and absence of mannans, we obtained ten binding indices for each sample. These indices form the foundation for our fingerprint analysis.

The volume of the reaction mixture was 300 µL. The number of cells from BALF was about 1 × 10^5^. The concentration of the IR marker was 1 mg/mL by PEG (equivalent to 0.2 mM), mannan 1 mg/mL. PBS (0.01 M, pH 7.4). T = 37 °C. Fluorescence of FITC was detected using a SpectraMax M5 device (Molecular Devices Corporation, California, USA) in the Costar black/clear-bottom tablet (96 wells) at λ_exci,max_ = 480 nm; λ_emi_ = 520 nm. The binding index was determined in % for a cell-bound polymer based on the difference between the initial and final fluorescence values.

#### 2.6.5. Agglutination of Cells from BALF—Turbidimetric Method

The kinetic curves of cell agglutination from BALF cells (10^6^ cells/mL) were constructed based on the relationship between A_600_ and incubation time, with mannan serving as the agglutinating agent. The turbidity of the system was measured using an Ultrospec 2100 Pro device (AmerSham Biosciences, Cambridge, UK). The medium used was PBS at a concentration of 0.01 M with a pH of 7.4, and the temperature was maintained at 37 °C.

### 2.7. Confocal Laser Scanning Microscopy (CLSM) of Eukaryotic Cells

The CLSM images of macrophages extracted from the BALF, model macrophages derived from human monocytes THP-1, and control non-phagocytic fibroblast cells lacking CD206 were obtained using the CLSM Olympus FluoView FV1000 (Olympus Corporation, Tokyo, Japan) equipped with both a spectral scan unit with emission detectors and a transmitted light detector. The motorized inverted microscope Olympus IX81 (Olympus Corporation, Tokyo, Japan) was employed. The objective-lens Olympus UPLSAPO 40× NA 0.90 (Olympus Corporation, Tokyo, Japan) was utilized for the experiments. The laser power, sampling rate, and averaging were identical for all image acquisitions. The scan area was 80 × 80 µm^2^. The cell nuclei were stained with DAPI—blue channel (λ_ex,max_ = 405 nm; λ_emi_ = 420–480 nm); the CD206 receptors on macrophages were stained with Alexa594+ antibodies—red channel (λ_ex,max_ = 594 nm; λ_emi_ = 620–700 nm); the fluorescent markers contain FITC label (PEI-triMan-FITC and PEI-Man-FITC)—green channel (λ_ex,max_ = 488 nm; λ_emi_ = 505–555 nm). The signals were adjusted to the linear range of the detectors. Olympus FV10 ASW 1.7 software was employed for the acquisition of the images.

## 3. Results and Discussion

### 3.1. Design of the Work

In this study, we propose a new approach to the diagnosis of respiratory tract disorders, with the potential for subsequent optimization of treatment strategies, based on a fingerprint method of bronchoalveolar lavage fluid (BALF) analysis. We present the concept and its practical implementation through the investigation and classification of macrophages from the BALF, which exhibit varying levels of the expression of mannose CD206 receptors. For the study, BALF samples were obtained from patients with diverse diagnoses, which differed in terms of their clinical course, such as acute, chronic, etc.

The classification of macrophages was achieved through the use of polymers with diverse carbohydrate moieties that exhibit varying affinities for CD206 receptors (Table 1). This process is based on the complicated molecular architecture of ligands comprised of galactose, mannose, or trimannose units, as well as mannan polymers (Figure 1). Different factors such as the overall length of the polymer chain, the distance between the saccharide residues, the arrangement of mannose residues as regular or random, and the structural characteristics of the polymer chain all contribute to the affinity for CD206 receptors. The binding of these ligands to macrophages is determined by the specific interaction between the ligand and a single receptor, as well as the receptors’ expression degree, their spatial arrangement, and potential clustering, allowing for a unique “fingerprint” analysis of macrophages that can be associated with specific diseases.

Key aspects of this work include:

1. Development and characterization of IR and fluorescent markers specific for CD206+ macrophages.
2. Classification of macrophages using FTIR spectroscopy in various modes: kinetic (real-time) analysis, pre- and post-binding comparison, and FTIR mapping of BALF samples.
3. Macrophage typing using fluorescent markers as an approach to fingerprint analysis of macrophage populations from BALF provides valuable insights into the diagnostics
4. Confocal visualization of macrophages from BALF, model macrophage cell lines, and CD206-negative cells.

### 3.2. Synthesis and Characterization of Infrared (IR) and Fluorescent Markers for Macrophage Typing

#### 3.2.1. Infrared (IR) Markers for CD206 Receptors

The principal concept of an IR marker was introduced by us in previous work [45] and involves a molecular probe for the infrared range. We suggest using polyethylene glycol (PEG) as one of these IR markers. The IR marker exhibits a distinct and analytically relevant spectral band at 1090 cm^−1^, which is distinct from the main functional groups characteristic of biopolymers such as hydroxyl (–OH), carboxyl (–COOH), amines, aromatic compounds, amides, and phosphate groups. The methylene (CH_2_) groups present in the composition of the IR marker structure can serve as reference peaks when analyzing PEG.

**Figure 2:**
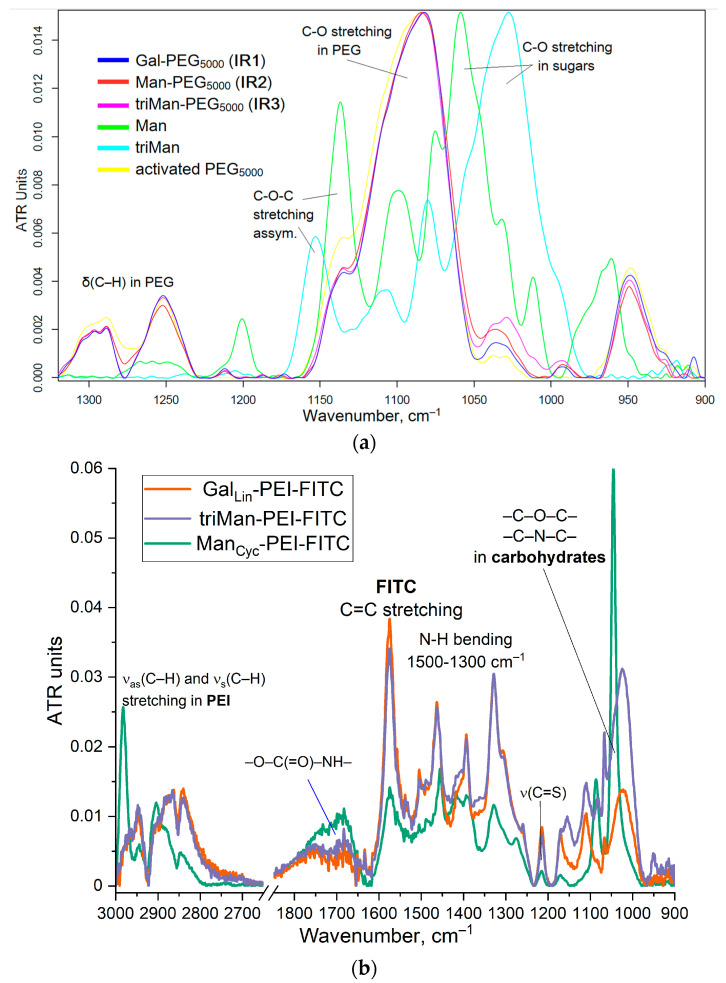

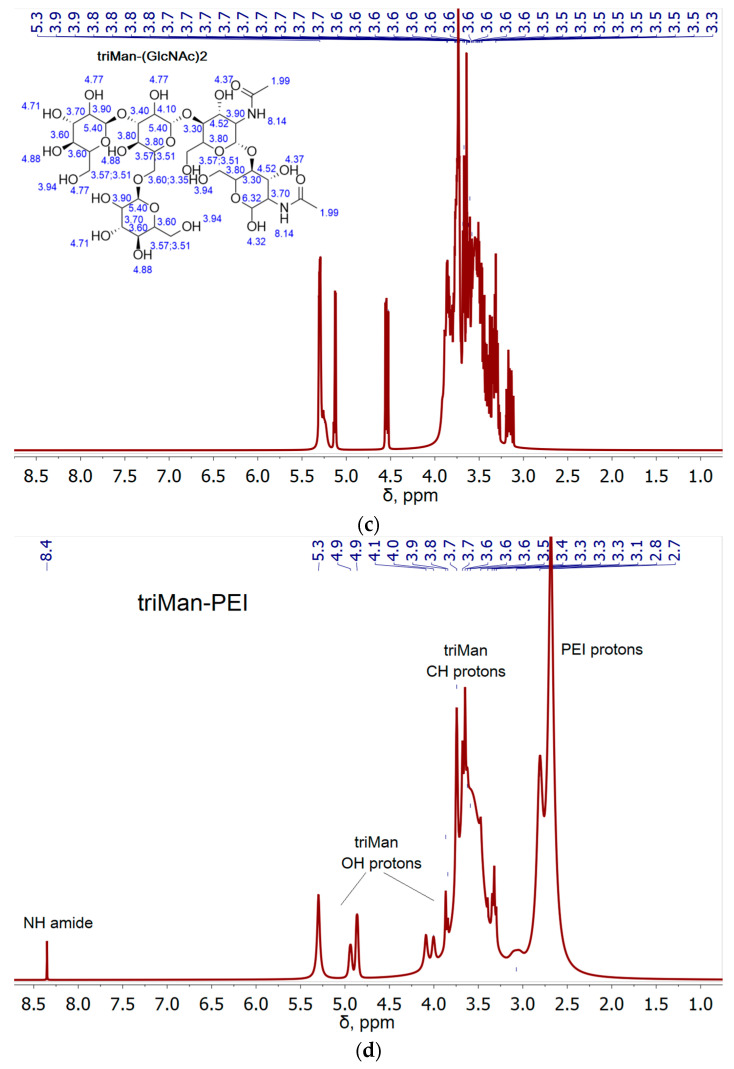

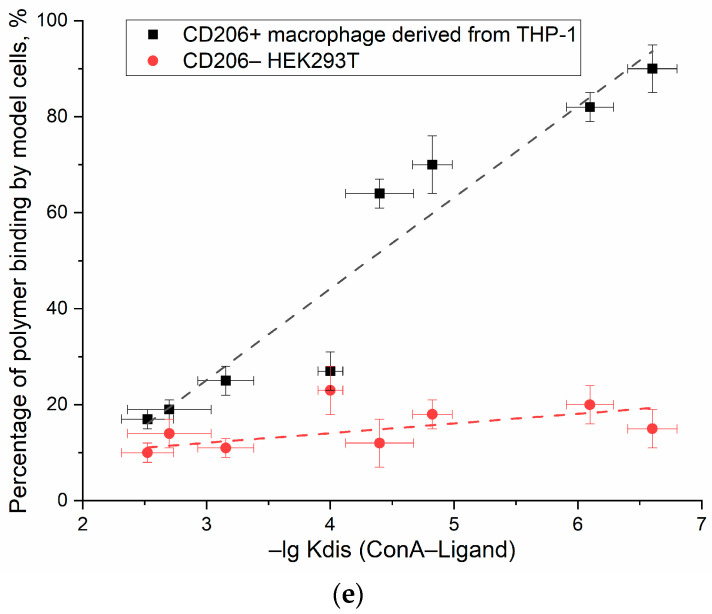
(**a**) FTIR spectra of reagents and products of synthesis of IR markers based on PEG and three types of carbohydrate fragments with different affinity to CD206 macrophage receptors. (**b**) FTIR spectra of fluorescent markers with different affinity to CD206 macrophage receptors X-PEI-FITC. (**c**) ^1^H NMR spectra of triMan-(GlcNAc)_2_. D_2_O. T = 22 °C. (**d**) ^1^H NMR spectra of triMan-PEI as a polymer for fluorescent markers. D_2_O. T = 22 °C. (**e**) Correlation of ligand affinity to the model mannose receptor ConA (Kdis for ConA–Ligand, M) with percentage of polymer binding by model cells. PBS (0.01 M, pH 7.4). T = 37 °C.

The synthesis of IR markers with three types of carbohydrate tags is illustrated in Figure 1a. The successful synthesis of PEG-carbohydrates is confirmed by the presence of a sharp and pronounced peak at 1090 cm^−1^ (C–O–C) in the FTIR spectra of the products, as shown in Figure 2a. Additionally, a minor shoulder at 1030 cm^−1^ is observed, corresponding to the ν(C–O) bond oscillations in carbohydrates. Furthermore, the formation of amide bonds is supported by the emergence of peaks at 1660 cm^−1^ ν(C=O) and 1580 cm^−1^ δ(N–H), replacing the peak typically observed at 1710 cm^−1^ ν(C=O) for activated PEG ethers (Figure S2a).

NMR spectroscopy provides complementary information about the structure of polymers. An example of the ^1^H NMR spectrum of Man-PEG is presented in Supplementary Figure S2b. The 5 kDa PEG polymer chain exhibits a series of overlapping proton signals of methylene groups in the 3.5 ppm region. Signals of mannose (or other saccharides) in the NMR spectrum appear at 4.2–4.6 ppm (OH groups), 3.45–3.6 ppm (CH–OH), and 2.8–3.0 (CH_2_), which confirms the formation of a linear shape and crosslinking with putrescine. Putrescine protons, in turn, produce a signal at 1.4–3 ppm depending on the location of the CH_2_ group. Thus, NMR spectroscopy confirms the structure of polymer IR markers.

#### 3.2.2. Fluorescent Markers for CD206 Receptors

Fluorescent ligand markers can be considered a viable alternative to antibodies: while the number of antibodies to the receptors is limited, the polymeric ligands can be synthesized in unlimited numbers by variations in the molecular architecture and composition. This allows for a significant expansion of the number of parameters in fingerprint analysis. With the application of a wide range of labels characterized by distinct affinities, one can conduct a fingerprint analysis, examining the binding patterns of macrophage cells with the set of ligands.

The process of fluorescent marker synthesis incorporating five types of carbohydrate tags is exemplified in Figure 1b. Figure 2a presents the infrared spectra of fluorescent markers derived from PEI with modifications incorporating linear galactose, cyclic mannose, and trimannoside residue. The spectra reveal pronounced oscillatory bands of CH_2_ groups in the PEI region spanning from 2980 to 2800 cm⁻^1^. The characteristic peaks at 1580 and 1450 cm⁻^1^ correspond to the valence oscillations of C=C in the fluorescein isothiocyanate (FITC) label. Peaks ranging from 1200 to 1000 cm⁻^1^ are associated with oscillations of C–N in the PEI molecule and C–O–C bonds in the saccharide components.

The ^1^H NMR spectra confirm the successful synthesis of polymers as a template for the creation of fluorescent markers. Figure 2c,d provides a visual representation of the NMR spectra for triMan and triMan-PEI. Key features observed include characteristic signals for mannose (H1, H2–H5, 3.9–5.3 ppm) or other sugars like glucose, confirming the carbohydrate moieties. The appearance of signals around 2.5–2.9 ppm confirms the presence of the PEI polymer, indicating successful conjugation between triMan and PEI—similar observations are made for other conjugates. Slight shifts in the carbohydrate proton signals (e.g., H5, 5.1–5.3 ppm) suggest a conformational change upon conjugation, consistent with either a linear or cyclic configuration of the reducing end of the oligosaccharide, as expected based on the reported literature [50]. The 1H NMR spectra of the Gal-PEI and Man-PEI conjugates show signals for both the carbohydrate and PEI parts of galactose protons in the range of 3.5–4.5 ppm, without the signal of anomeric protons (H-1) at about 5–5.5 ppm after sugar modification. Mannose signals are similar to PEI signals and appear in the region of 2.5–2.9 ppm. 

Thus, the use of Gal-PEI and Man-PEI conjugates can serve as a basis for creating fluorescent markers that will allow the accurate identification of CD206+ macrophages. This is a promising area that can improve the diagnosis and treatment of various diseases of the body.

#### 3.2.3. Physico-Chemical Parameters of IR and Fluorescent Markers Used for Macrophage Typing

Table 1 presents the physicochemical attributes of the investigated markers. The IR markers are distinguished by an equimolecular ratio of PEG chains and saccharide substituents. The particles comprising the IR markers exhibit an average hydrodynamic diameter of approximately 85–90 nm and are characterized by slightly positive values of the zeta potential.

The trisaccharide moiety of IR3 exhibits a pronounced affinity for CD206 mannose receptors, whereas the galactose ring configuration of IR1 manifests a weak affinity to CD206 receptors. The linear mannose component displaying the lowest affinity to CD206 (Table 1) and present in IR2 functions as a negative control.

Fluorescent markers are engineered based on PEI with a molecular weight of 1800 Da, which are grafted with FITC residues at a frequency of one per polymer chain and carbohydrate residues ranging from 10 to 18 per 1 polymer chain. The particles of these fluorescent markers exhibit an average hydrodynamic diameter in the range of 100–150 nm and possess positive zeta potentials. One such marker, specifically designated for high-affinity labeling of the CD206 receptor, is triMan-PEI-FITC. Medium-affinity labels for the CD206 receptor are Man_Cyc_-PEI-FITC and Gal_Cyc_-PEI-FITC, with cyclic sugar residues, while control polymers with low affinity contain linear residues of galactose and mannose.

For the quantitative determination of the affinity of mannose ligands for the mannose receptor, we used concanavalin A (ConA) lectin as a model reference receptor (Figure 2e and Table 1). There is a positive correlation between the dissociation constant of the ConA-ligand and the binding degree to the CD206+ macrophage cells. In contrast, for CD206-negative control cells, the binding was largely independent of the marker type, indicating non-specific binding.

The interaction of IR and fluorescent markers with BALF macrophages is based on several key mechanisms:

- Primary ligand–receptor interactions [12,13,14,15]: IR and fluorescent markers, being mannosylated or glycosylated polymers, can bind to macrophage surface receptors. These receptors include lectins (CD206 [16,17,18], CD301 [19,20], etc.) or other proteins specific to mannose or other sugars.
- Non-specific electrostatic binding [21,22,23]: Cationic polymers, such as PEI, can interact with negatively charged sites on the macrophage surface due to electrostatic forces. This binding can be controlled by carrying out the binding experiments and washing the samples with solutions with ionic strength (0.15 M NaCl) to avoid the retention of the polymer on the cell surface.
- Phagocytosis [26,27,28]: After the initial binding of IR and fluorescent markers, macrophages can trigger receptor-mediated phagocytosis to absorb these particles. These processes can lead to various biological effects, such as the activation of macrophages or modulation of the immune response.

### 3.3. The Methods of Analysis Macrophages from BALF and Diagnosis Aspect

#### 3.3.1. FTIR Spectroscopy

By examining the interactions between macrophages and specific markers, we can gain insights into the functional characteristics of these cells. This information is crucial for gauging the severity of the disease, facilitating accurate diagnosis, and optimizing treatment strategies.

In the context of investigating macrophages using FTIR spectroscopy, we launch three experimental approaches that offer complementary insights:

1. Real-Time Experiment. This method enables the observation of alterations in the absorption spectrum resulting from the interaction of mannose and other carbohydrate-based ligands with macrophages. We could analyze the kinetics and efficacy of this binding process. The kinetics and extent of ligand attachment parameters are directly related to both the polarization state and phagocytic capacity of macrophages.
2. IR spectra before and after the ligand binding are of particular interest, providing information on the state of the ligand inside the macrophages. After a few hours of incubation, the phagocytic activity can be assessed. We can estimate the ligand content internalized inside the macrophages by receptor endocytosis and identify the specific organelles or biomolecules involved in the interaction.
3. IR micro-mapping. Application of the infrared imaging technique provides detailed information on the precise distribution of mannose ligands on the surface of macrophages. This approach allows for the visualization of regions where binding sites are present and the quantification of the strength of these interactions.

#### 3.3.2. Real-Time FTIR Experiments for Macrophage Typing and Disease Diagnosis

**Figure 3:**
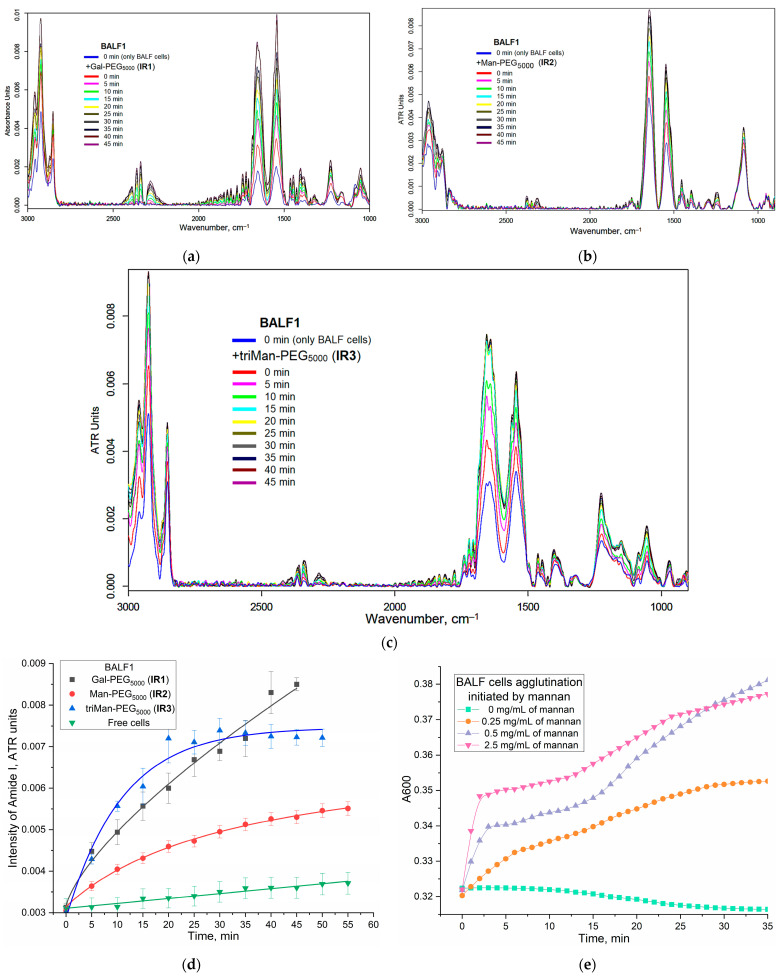
FTIR spectra of BALF samples (from a patient with purulent endobronchitis) during real-time incubation with IR markers: (**a**) IR1; (**b**) IR2; (**c**) IR3 of different affinity to CD206 receptors. (**d**) Kinetic curves of the intensity change in the Amide 1 peak at graphs (**a**–**c**). The volume of the reaction mixture is 35 µL. The number of cells from BALF is about 2 × 10^5^. The concentration of the IR marker is 5 mg/mL by PEG (1 mM). (**e**) Kinetic curves of agglutination of BALF cells (10^6^ cells/ mL) according to the dependence of A600 on incubation time with mannan as the initiator. Control graph for item (**d**). PBS (0.01 M, pH 7.4). T = 37 °C.

Figure 3 shows the FTIR spectra of BALF samples (from a patient with purulent endobronchitis) during real-time incubation with IR marker IR1-3 of different affinity to CD206 receptors. The characteristic peaks of cells in the IR spectra are the following bands: ν(CH_2_) (3000–2800 cm^−1^, lipid membrane), ν(C=O) (Amide 1, 1700–1600 cm^−1^, proteins), δ(N–H) (Amide 2, 1600–1500 cm^−1^, proteins), ν(P=O) (1300–1200 cm^−1^, DNA), and ν(C–O) (1100–1000 cm^−1^, carbohydrates). When IR markers containing PEG chains interact with cells, polymer binding to the cells and their subsequent deposition are observed, which is evident from an increase in the intensity of all major peaks. The rate of deposition is particularly high in cases of strong affinity binding.

We examined the interaction between polymers and macrophages «in adsorption and binding mode» by using the amide 1 region (corresponding to interaction with protein CD206-receptor). The increase in the intensity of the amide signal is associated with the conformational adjustment of the receptor protein and its engagement with the triMan-PEG microenvironment that specifically binds to the CD206 receptor. This interaction subsequently results in a heightened amide peak. In our previous study, we encountered a similar phenomenon when investigating the model protein ConA and its specificity toward carbohydrate and mannose ligands with varying structures. It was observed that the greater the affinity of a ligand, the more pronounced the increase in the Amide 1 peak [20,21,51].

Figure 3d presents the kinetic curves of the Amide 1 peak intensity on the incubation time of the ligand with macrophage cells. Besides the conformational adjustment of the receptor protein upon interaction with the mannose ligands, we observe the cellular agglutination of BALF macrophages as they adhere to carbohydrate and polymer ligands. Turbidimetric analysis also revealed the agglutination of macrophages in their interaction with highly specific mannose ligands (Figure 3e). This phenomenon becomes more evident as the affinity of the label for CD206 receptors intensifies, while the contribution of background cellular deposition of cells (without ligands) can be disregarded.

In the case of IR3 (triMan-PEG), there is an initial sharp increase followed by saturation after 20 min. When using IR2, containing linear mannose, the binding is not as pronounced compared to triMan-PEG. This indicates the specific nature of binding and suggests that the macrophages are CD206+, indicating the activated state of macrophages. This observation is consistent with the diagnosis of purulent endobronchitis. We present some examples of the graphs of this particular technique.

As an alternative analysis approach, we applied the normalizing spectra to the Amide 1 (protein) peak and analyzed the bands assigned to PEG (in IR-label) so that we could specifically track the binding of polymers to cells.

**Figure 4:**
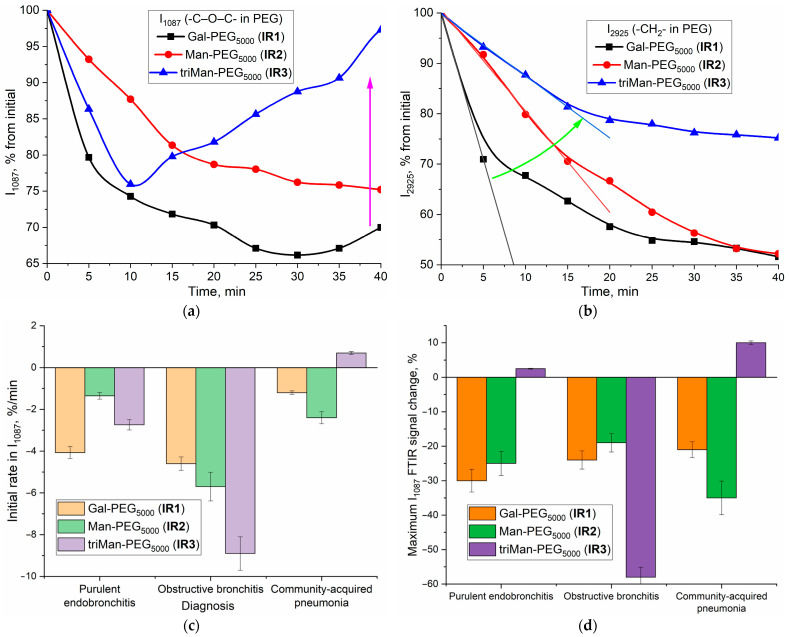
(**a**,**b**) Kinetic curves of the intensity change in the peak at 1087 and 2925 cm^−1^ in normalized on Amide 1 peak FTIR spectra of BALF samples (from a patient with purulent endobronchitis) during real-time incubation with IR markers. The conditions are similar to those shown in Figure 3. The green line shows the change in the initial section of the kinetic curves when the affinity of the ligand changes. (**c**,**d**) Macrophage binding indices from BALF (initial velocity and maximum change in IR spectra) using the real-time FTIR experiment method and its relationship with patient diagnoses. Several BALF samples were used for experiments, with one patient and one sample corresponding to one diagnosis; however, several repeated measurements were provided for each sample (n = 3).

Analytically significant peaks for IR-label (PEG) can be observed at 1087 cm^−1^ (C–O–C, PEG) and 2925 cm^−1^ (CH_2_, PEG and the lipid membrane of the cells). In BALF obtained from a patient with endobronchitis, a distinct pattern of polymer binding to macrophages emerges (Figure 4a,b). For the peak at 1087 cm⁻^1^, the initial rate is highest for IR1 (linear galactose, −30% for 10 min). In the case of the most affine ligand IR3 containing triman, we observed rapid quenching (−26% within 10 min) of the PEG signal due to the binding with the receptor protein, and a subsequent “ignition” effect (+25% over a 30-min period) upon specific interaction between the polymer and cells, resulting in receptor endocytosis. This manifested as an increase in signal intensity attributed to changes in the microenvironment upon internalization of CD206 receptors upon interaction with high-affinity polymers.

For the peak at 2925 cm^−1^ (Figure 4b), the kinetic curves are characterized by a hyperbolic trend with saturation. However, the C-O-C peak is more sensitive to the binding of macrophages to ligands.

Based on a set of analogous graphs, we have determined the indexes of polymer binding to macrophages for several clinical conditions (Figure 4c,d): purulent endobronchitis, obstructive bronchitis, and pneumonia. The quantification of macrophage polarization in BALF can be accomplished through the selectivity index, determined as the ratio of ligands of different affinities binding to cells. Each condition exhibits its own distinctive index pattern, corresponding to the specific clinical presentation and the state of macrophage polarization.

To assess the polarization state of macrophages in BALF (bronchitis with a moderate degree of inflammation), we correlated the binding indices determined for BALF with the control test systems using the flow cytometry method (Table 2). As a positive control, we used a model monoclonal cell line derived from THP-1 monocytes that exhibited a highly homogeneous phenotype M1 and characterized by high expression of CD206 receptors. The binding indices determined for homogeneous M1 were compared to those for macrophages from BALF. CD206-negative HEK293T cells were employed as the negative control.

In the case of a model cell line (in M1 state) of activated macrophages, triMan-PEI appears to have the highest affinity to the CD206 receptor, cyclic mannose and galactose exhibit moderate binding efficiency, and linear galactose is non-specific and exhibits the lowest affinity for the mannose receptor in M1 macrophages. The resulting selectivity index determined the maximal binding ratio of triMan-PEI to Gal_Lin-_ PEI of 3.6.

In the case of HEK293T, binding to carbohydrates is relatively weak, and no specificity is observed.

In the case of macrophages from the BALF of patients with inflammation, selective binding for the highly affined ligand also emerges, indicating the activation status of macrophages. The resulting selectivity index determined the maximal binding ratio of Mannan- to MAN_Lin_- PEI of 4.6. BALF cells show slightly different specificity (Mannan ≈ triMan > Gal_Cyc_ >Man_Cyc_ >> Gal > Man) compared to model macrophages (triMan ≈ Mannan >> Man_Cyc_ ≈ Gal_Cyc_ >> Man >> Gal), which indicates a different distribution of macrophage subpopulations M1, M2, and its subtypes.

In our IR test with PEG based-IR-labels for the case of purulent endobronchitis (Figure 4c,d), we observe a high specificity index (affine non-specific ligand), corresponding to the activated state of the macrophages. Moreover, we observe a higher affinity for galactose than for the mannose-based ligand and a medium binding level, i.e., the features are quite different from the model M1 phenotype (Table 2). This demonstrates the sensitivity of the IR test with PEG-based IR labels to detect a multitude of macrophage subpopulations, rendering it crucial in the application of the fingerprint method for macrophage polarization analysis.

In the case of obstructive endobronchitis, we also observe a high specificity index (affine non-specific ligand) and a higher affinity for mannose than for galactose and a high binding level, i.e., patterns are different from the M1 control system and correspond to a mixture of M1 and M2 with a prevalent M2-like phenotype (with its subpopulation).

In the case of pneumonia, we observe a high specificity index (affine non-specific ligand) and a comparable affinity for mannose compared to the galactose ligand, i.e., an approximately similar pattern to the M1 model macrophage (Table 2), which is consistent with the theoretically high level of CD206 expression according to the established M1-like phenotype of macrophages.

#### 3.3.3. “Before and After” FTIR Experiments for Macrophage Typing and Disease Diagnosis

##### Spectral Changes

**Figure 5:**
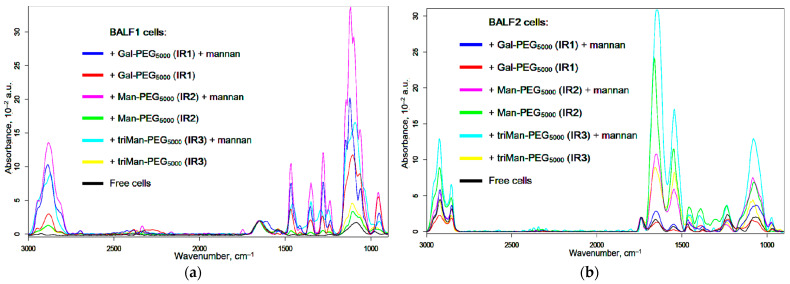

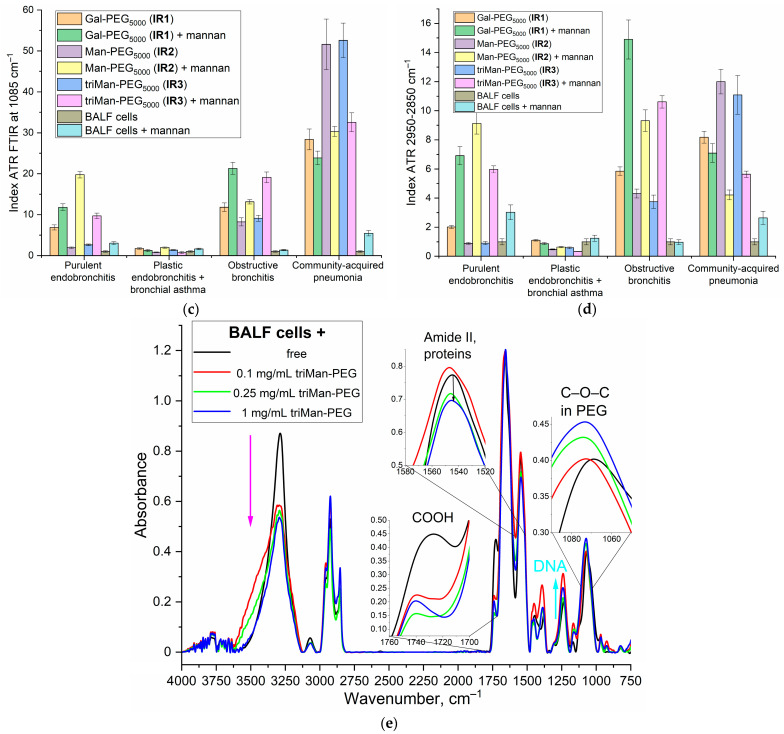
FTIR spectra of BALF samples after 2 h incubation with IR markers and mannan with subsequent washing of the unbound polymer. (**a**) BALF samples from a patient with purulent endobronchitis. (**b**) BALF samples from a patient with bronchial asthma (with plastic bronchitis). (**c**,**d**) Macrophage binding indices from BALF (relative intensity of characteristic peaks in FTIR spectra) using the «Before and After» FTIR experiment technique and its relationship with patient diagnoses. The volume of the reaction mixture is 300 µL. The number of cells from BALF is 5 × 10^5^. The concentration of the IR marker is 2 mg/mL by PEG (equivalent to 0.4 mM), mannan 2 mg/mL. (**e**) FTIR spectra obtained from BALF samples from a patient diagnosed with bronchiectasis and bronchial asthma following a 6 h incubation period with different concentrations of the triMan-PEG IR marker followed by the washing from an unbound polymer. PBS (0.01 M, pH 7.4). T = 37 °C.

An alternative to the real-time analysis of macrophage interactions with polymers is the «Before and After» technique of FTIR experiments, allowing us to assess the amount of polymer internalized into the cells. Figure 5a,b illustrates the IR spectra of BALF samples from patients with purulent endobronchitis and bronchial asthma (with plastic bronchitis). Cell samples were incubated with IR1–IR3 polymers (with different affinities) for 3 h, either in the presence or absence of mannan—as a concurrent agent for binding with the CD206 receptor. The amount of bound and internalized polymer can be determined from the intensity of spectral peaks at 2950–2850 cm^−1^ (CH_2_ group) and 1087 cm^−1^ (C–O–C). The interaction of BALF cells with polymers leads to an increase in the intensity of characteristic peaks, especially v(C–O–C), which indicates adsorption of the polymer on the cell wall as well as receptor-mediated endocytosis. Figure 5c,d illustrates the binding indices of BALF cells to different polymeric ligands. By examining the ratios and magnitudes of these indices, it becomes possible to differentiate between acute and non-acute diseases and chronic and local conditions, among other distinctions.

##### Diagnosis

In the case of **purulent endobronchitis**, an increase in intensity of PEG-IR marker peaks (at 2950 and 1087 cm^−1^) was observed in BALF macrophages upon ligand binding. Again, a higher affinity for galactose than the mannose-based ligand is observed. This feature is different from the model M1 phenotype but similar to model BALF (from Table 2) corresponding to bronchitis with a moderate degree of inflammation. Interestingly, in the case of **acute illnesses**, such as **purulent endobronchitis and pneumonia**, in the presence of mannan, a marked synergistic effect in the ligand binding is observed: the interaction between markers and CD206 receptors is enhanced. In other words, mannan acts not as a binding inhibitor but rather as an effector in this context. The interaction of mannan with CD206 receptors can be interpreted as follows: the presence of mannan enhances the efficiency of ligand binding to CD206 due to the cooperative effects and increased affinity of the receptors for carbohydrate ligands, resulting in optimal receptor configurations. This is reasonable as one of the natural functions of CD206 is to engage in the binding of microorganisms that possess mannose patterns. When oligo- or polymannosides are bound, the binding mechanism is activated. For instance, the highest binding indices are observed in cases associated with bacterial infections, such as pneumonia with an acute course. Conversely, for chronic conditions such as **plastic endobronchitis with elements of bronchial asthma**, the levels of all biomarkers are quite low. This suggests a small number of activated macrophages with a tendency toward M2-like polarization. Therefore, the acquired data are feasible to categorize BALF cells and establish an inflammation degree of the respiratory tract disease.

##### Phagocytosis

Phagocytosis represents a crucial aspect of the interaction between BALF cells, particularly macrophages, and IR markers. This process becomes evident when polymer samples are incubated with cells for more than four hours.

Figure 5e illustrates the IR spectra of BALF samples from a patient diagnosed with bronchiectasis and bronchial asthma after a 6 h incubation period with different concentrations of the triMan-PEG IR marker. After the incubation, the BALF samples were washed from the unbound polymer. Changes in the IR spectra after receptor phagocytosis were detected, following a comparative analysis of the spectra obtained from cells with and without polymers:

1. The intensity of the peak at 1730 cm^−1^, corresponding to the COOH group of the cell, decreased due to the interaction with polymers and the subsequent shielding effect on the BALF cells.
2. The intensity of the amide 2 (protein) peak decreases with the increase in the polymer concentration, suggesting the penetration of polymers into the cells through phagocytosis.
3. There is an increase in intensity, accompanied by a shift in the high-frequency region of the peak at 1070 cm^−1^ (C–O–C in PEG), which is attributed to a modification in the microenvironment of the PEG chains during the process of phagocytosis. About 20% of the polymer was internalized after being incubated for 6 h with macrophages.
4. The intensity of the peak grows by 1250 cm^−1^, corresponding to phosphate groups in DNA, indicating an interaction between the polymer and macrophage DNA of macrophages.

These alterations in the IR spectrum provide evidence of the progression of phagocytosis in the presence of high polymer concentrations after a 6 h incubation period, thereby confirming the efficacy of the employed polymers for macrophage identification.

#### 3.3.4. FTIR Mapping (Microscopy) of BALF Samples with Polymers

FTIR spectroscopy offers a diverse array of techniques and functionalities for monitoring the interaction between BALF cells and polymers, including the utilization of FTIR mapping methodology, commonly known as (FTIR microscopy). The advantages of this approach are manifold: non-destructive analysis on the cellular level, enabling the generation of images depicting the distribution of chemicals within a sample; FTIR microscopy exhibits exceptional sensitivity to chemical groups and molecules, enabling the detection of even minute alterations in their distribution within the sample. This sensitivity makes it possible to precisely pinpoint the binding sites of cells to polymers.

Furthermore, this method provides the ability to investigate the mapping of the components of cell membranes, polymers, and other substances, which can prove invaluable in diagnosing and monitoring various diseases.

**Figure 6:**
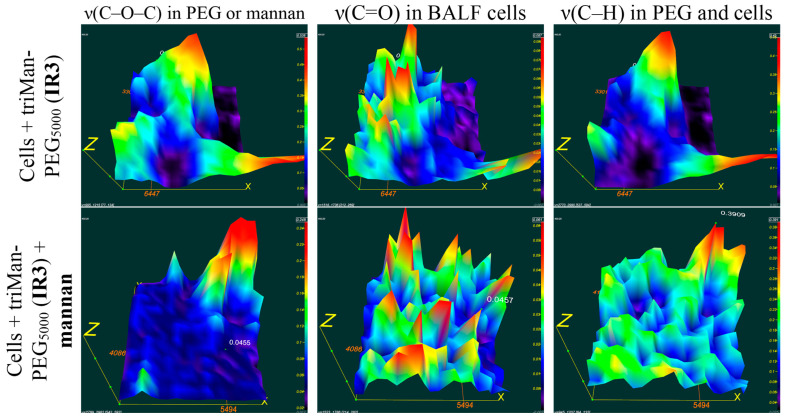
FTIR microscopic maps of the peak intensities’ integral distribution in the IR spectra of BALF samples (diagnosed with purulent endobronchitis) incubated for 1 h with triMan-PEG_5000_ (10 mg/mL) in one version followed by mannan treatment (10 mg/mL) for 1 h. PBS (0.01 M, pH 7.4). T = 37 °C.

Figure 6 presents FTIR microscopic maps of the integrated distribution of intensities within the spectra of bronchial alveolar lavage fluid (BALF) samples obtained from patients diagnosed with purulent endobronchitis. The samples were incubated with triMann-PEG_5000_, followed by treatment with subsequent mannan treatment and without mannan.

It is crucial to examine the colocalization of IR signals from cells and the polymer as it would indicate the binding of the polymer to the cells. For triMan-PEG5000, the distribution patterns of cells (represented by yellow-red maxima corresponding to peaks v(C=O), indicating single or clustered cells from BALF) closely resemble the distribution of PEG (at peaks v(C–O–C)), suggesting a strong affinity of IR markers bearing a trimannose substituent for these macrophages.

In the presence of mannan, the treatment of a BALF sample with triMan-PEG_5000_, the distribution of the polymer is not colocalized with cells, suggesting a high level of competition for CD206 receptor sites. This inhibition effect implies specific binding with the CD 206 receptor and the activated state of the macrophages.

The data obtained through various techniques employing FTIR spectroscopy provide detailed information and complement each other. IR microscopic maps of BALF samples obtained from patients with purulent endobronchitis and bronchial asthma, including plastic bronchitis, are presented in Supplementary Figures S3 and S4. Based on a thorough comparison of co-localization patterns and the absence of signals from cells and polymers, similar to the above procedure, it can be concluded that in cases of bronchial asthma accompanied by plastic bronchitis, the state of macrophage polarization is different from M1 model macrophages and tends toward M2 subpopulations.

#### 3.3.5. Fluorimetric Typing of Macrophages Derived from the BALF

##### FITC Markers Binding to Model Cell Cultures and BALF Cells

As a complementary method, we suggest the fluorimetry test for the typing of macrophages derived from BALF for quantifying ligand–receptor interactions. Five fluorescent polymers labeled with FITC based on PEI with grafted carbohydrate moieties were tested for CD206 receptor affinities. The highly specific ligand is triMan-PEI-FITC. The medium-affinity labels for CD206 include Man_Cyc_-PEI-FITC and Gal_Cyc_-PEI-FITC, featuring cyclic sugar moieties. Control polymers with low affinities contain linear moieties of either galactose or mannose.

**Figure 7:**
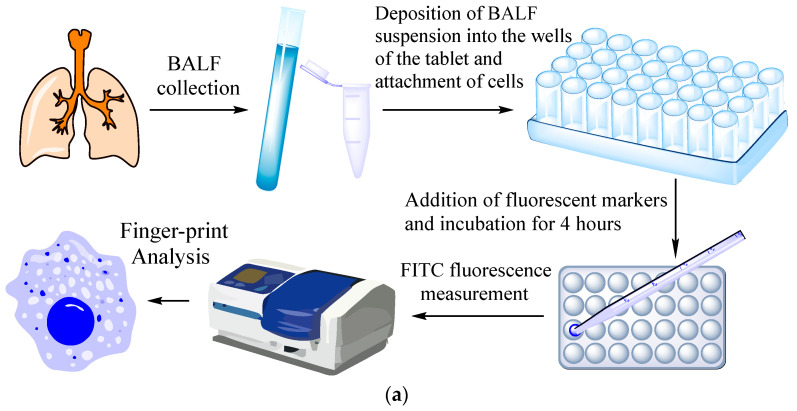

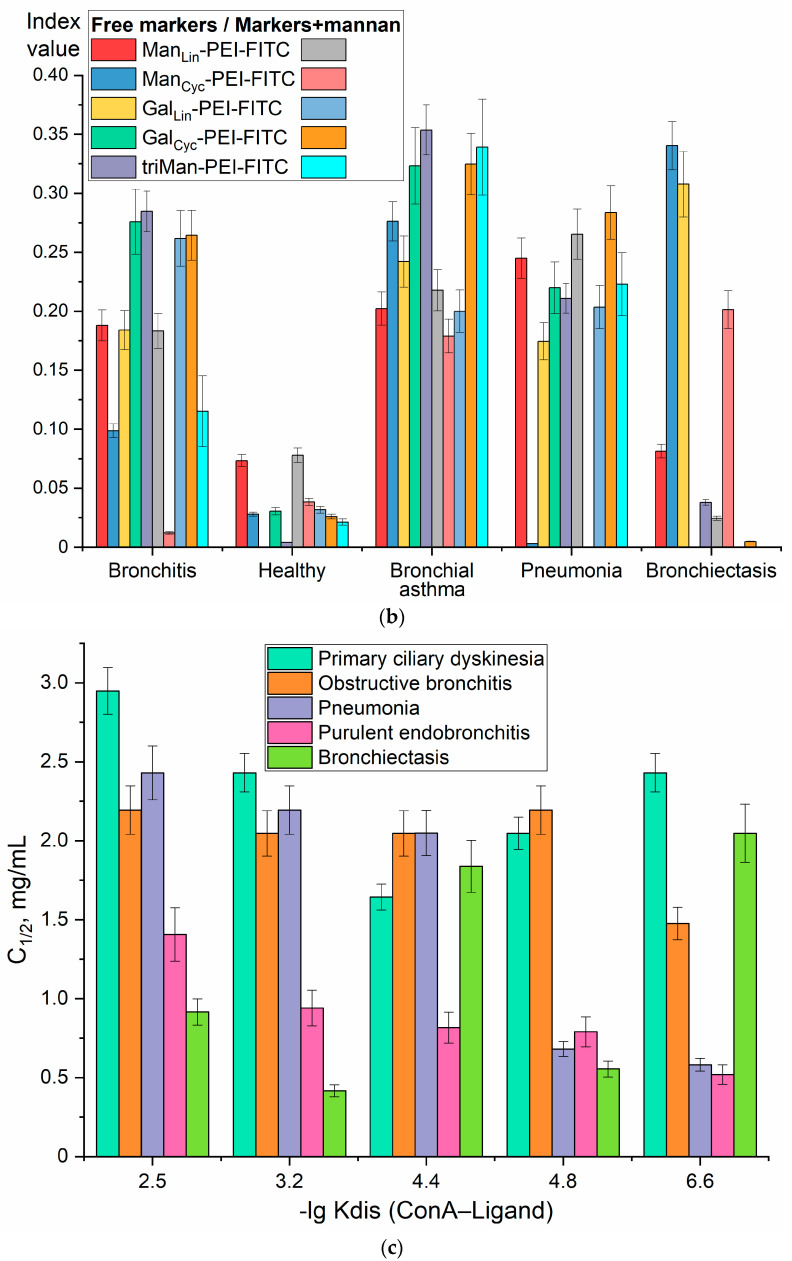
(**a**) Schematic representation of the experiment on fingerprinting analysis of macrophages from BALF in terms of polymer binding. (**b**) Binding indexes of fluorescent FITC-ligands with cells from BALF as a “fingerprint” of certain diseases. (**c**) The correlation between the dissociation constants of ConA with ligand and the ligand concentration of the half-maximal (C_1/2_) binding to BALF cells. The number of cells from BALF is 1 × 10^5^. PBS (0.01 M, pH 7.4). T = 37 °C.

The typical experimental procedure is presented in Figure 7a. Firstly, cells from BALF were isolated followed by deposition of the suspension in the wells of a plate, allowing cell attachment. Fluorescent markers were then added, followed by incubation for 4h. The fluorescence of the solutions was analyzed, and binding indices were calculated (the proportion of bound polymer). Employing a number of ligand markers, we derived ten sets of binding indexes for each BALF sample, forming the foundation of fingerprint analysis.

##### FITC Markers for Typing Macrophages from BALF and Correlating with Diagnoses

We examined a few case studies of disease conditions. For each disease, a distinctive set of binding indexes was obtained, akin to a “fingerprint” (Figure 7b). The binding index is presented as a quantitative measure of the bound ligand proportion. Values ranging from 0 to 0.1 are considered negligible, indicating an insignificant level. The range between 0.1 and 0.25 represents an elevated marker level. Values from 0.25 to 0.5 indicate a high level of marker presence. Finally, values exceeding 0.5 are indicative of a very high level of the marker.

In the case of a **healthy individual**, the index values fall within the range of 0.05–0.10 units, representing the norm. So, activated macrophages are practically absent in a healthy individual. Conversely, for diseases, there is a notable increase in these indexes.

For instance, in the cases of **bronchitis and asthma**, the binding indexes associated with the binding of cyclic galactose and trimannose exhibit a marked rise (up to 0.3–0.4 units).

In the case of **bronchiectasis**, a peculiar pattern emerges: low index values of six markers, with relatively elevated indexes corresponding to the binding of linear galactose and mannose, standing out as a distinct feature. The most highly affine triMan-PEG ligand binds most in the case of bronchitis and asthma and less than in the case of pneumonia, which complements the IR spectroscopy data.

To quantitatively characterize macrophage polarization status, we determined the concentration of a ligand at which half-maximal binding to BALF cells occurs (C_1/2_ parameter), serving as a «fingerprint» for specific diseases (Figure 7c). We established a correlation between the affinity of fluorescent probes for CD206 receptors on macrophages using the ConA (model CD206) and the C_1/2_ parameter, indicating that the activation status of macrophages is directly linked to the overexpression of CD206 (Figure 7c). Macrophages expressing CD206 exhibited lower C_1/2_ values for ligands with higher affinity (Figure 7c), a phenomenon particularly evident in individuals diagnosed with pneumonia or purulent endobronchitis, where CD206 assumes a prominent phenotype. These findings are in agreement with FTIR techniques.

#### 3.3.6. Confocal Imaging of the Interaction Between Polymer IR and Fluorescent Probes with BALF Macrophages, Model Cells of CD206+ Macrophage, and CD206− Fibroblast Cell Lines

To directly demonstrate that our FITC ligands (Table 1) specifically bind to CD206+ macrophages from BALF, we investigated the binding of polymer fluorescent markers to model cells, CD206+ macrophages (as positive control) vs. CD206− fibroblasts (negative control), to validate the selectivity of these markers.

**Figure 8:**
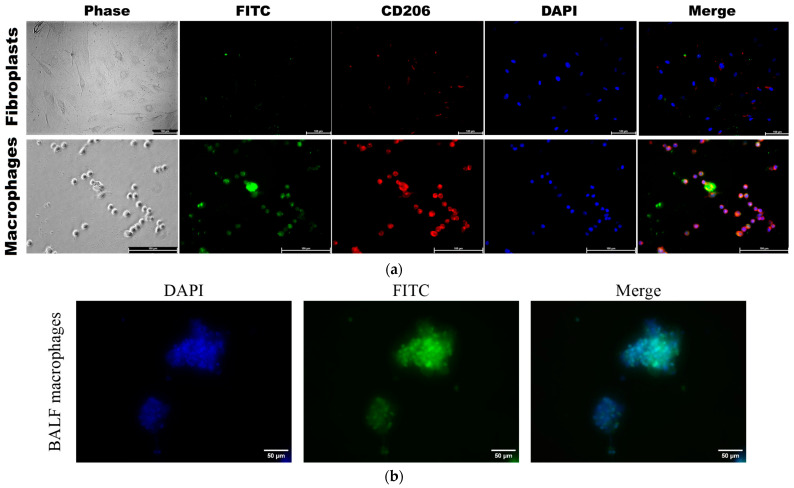
(**a**) CLSM images of THP-1-derived macrophage-like cells and human dermal fibroblasts (HDF). Binding assay with PEI-triMan-FITC after incubation for 40 min (green channel). Phase contrast microscopy, fluorescent microscopy, blue channel—nuclei stained with DAPI, red channel—CD206 receptors were stained with Alexa594. Scale bar, 100 μm. The full set of images is presented in Supplementary Figure S5. (**b**) Fluorescence images of BALF cells (macrophages). Binding assay with PEI-triMan-FITC after incubation for 40 min (green channel). Phase contrast microscopy, fluorescent microscopy, blue channel—nuclei stained with DAPI. Scale bar, 100 μm.

Figure 8a, available in its full version as Figure S5, presents CLSM images of CD206 + THP-1 macrophages derived from monocytes, along with control CD206− negative fibroblasts. The presence of bright red fluorescence in macrophages serves as a visual indication of CD206 expression, in contrast to the lack thereof in fibroblasts. The vivid green fluorescence observed in macrophages under analogical conditions serves as a clear indication of a markedly higher level of binding efficiency for CD206+ polymers compared to CD206− fibroblasts, with a selectivity exceeding 8–10 times according to cytometry. Confocal imaging reveals that mannosylated polymers exhibit a remarkable affinity for CD206+ macrophages.

Figure 8b presents fluorescence images of BALF cells, specifically macrophages, following 1 h incubation with PEI-triMan-FITC (green channel). The macrophages extracted from BALF exhibit a notable degree of heterogeneity, characterized by their larger size ranging from 20 to 40 microns, in contrast to the size of individual macrophages, which typically fall between 10 and 20 microns.

### 3.4. The Prospect of Typing Macrophages Derived from BALF for Medical Applications

The prospect of typing macrophages derived from BALF for medical applications is a promising avenue of research. This technique holds the potential for diagnosing and monitoring a wide range of conditions, including chronic diseases such as asthma, bronchiectasis, and chronic bronchitis and acute conditions like pneumonia and bacterial infections.

One approach to macrophage typing involves employing infrared markers to detect the binding of specific polymers by BALF-derived cells. Alternatively, fluorescent markers with varying affinities can be utilized to differentiate between macrophage populations. These techniques allow for the precise identification of macrophages and the creation of detailed profiles of macrophage subsets within individual patients. This has significant implications for early disease detection, tailored treatment strategies, and the development of personalized medical approaches.

Nonetheless, despite the promising potential, the employment of infrared (IR) and fluorescent markers in the process of classifying macrophages derived from bronchoalveolar lavage (BALF) necessitates further investigation. It is imperative to establish standardized methodologies and protocols for the analysis of data obtained through the use of these markers.

One avenue for BALF macrophage analysis involves manipulating their polarization and activation states for the treatment of a wide range of conditions, including infectious, autoimmune, and even oncological disorders. The polarization status of macrophages plays a critical role in resolving numerous inflammatory processes, therefore making it possible to identify key points within the body through the diagnosis of macrophage status.

Employing IR markers and fluorescent probes for the classification of macrophage subpopulations from BALF represents a promising approach with significant potential for medical applications in diagnostic procedures and as a means of modulating macrophage function.

### 3.5. Prospects for Using Polymer Ligands for New Methods of Treating Bronchial Asthma

Polymer mannosylated ligands are a promising tool for targeted therapy of asthma. Their ability to bind to mannose receptors on the surface of macrophages allows them to be used for two main approaches:

1. **Macrophage repurposing:** Mannosylated polymers can be used to deliver molecules that modulate macrophage or eosinophiles function. For example, the delivery of anti-inflammatory agents could switch the polarization of M1 macrophages (pro-inflammatory) to M2 macrophages (anti-inflammatory), reducing the inflammatory response in the lungs. This would reduce the symptoms of asthma, such as bronchospasm and inflammation of the lungs.
2. **Targeted drug delivery.** These ligands can serve as carriers for targeted drug delivery directly to macrophages or eosinophils, the main cells involved in the pathogenesis of asthma. This increases the effectiveness of the treatment and reduces side effects since the drug will be concentrated at the site of inflammation and not distributed throughout the body. This approach allows one to use smaller doses of drugs, reducing the risk of systemic toxicity of the drug.

Thus, polymer mannosylated ligands open up new opportunities for developing more effective and safe methods of treating asthma by targeting key cellular targets involved in the development and maintenance of the inflammatory process.

The prospects for using this technology are very promising. Targeting macrophages, key cells involved in inflammation conditions, could lead to more effective and safer medicines. The ability to modulate the immune response and reduce side effects makes this technology attractive for developing new treatments for asthma, potentially improving the quality of life of patients and reducing dependence on existing, often not sufficiently effective, therapeutic strategies.

## 4. Conclusions

Macrophages, present in bronchoalveolar lavage fluid (BALF), serve as valuable indicators for assessing the severity and predicting the course of respiratory illnesses. By analyzing their phenotypic and functional characteristics, we can discern unfavorable patterns associated with inflammatory, fibrotic, and tissue-remodeling processes. This approach provides novel insights into how the disease is likely to progress. Moreover, identifying markers of inflammation and fibrosis within macrophages from BALF enables us to evaluate the efficacy of treatment. Comparing macrophage profiles pre- and post-therapy allows us to assess their impact on inflammatory and fibrotic pathways. Here we have attempted to assess the differences between patient-derived macrophage populations from BALF samples in different pulmonary disease conditions, based on their capability to interact with a range of specific as well as relatively non-specific carbohydrate-based ligands (containing galactose, mannose, trimannose, etc.).

This study introduces a novel multiplexed approach for identifying macrophage subsets using a unique spectral fingerprint generated by carbohydrate-conjugated polymeric probes, significantly improving the sensitivity and specificity of macrophage phenotyping. The use of IR and fluorescent probes in fingerprint multiparametric analysis to categorize macrophages derived from BALF holds immense potential for medical applications, particularly in diagnostic procedures and manipulating macrophage function.

Here, we developed an expanded panel of ligands (with different specificities) for typing immune cells in bronchoalveolar lavage (BAL) of patients with bronchial asthma—with a special focus on patients with severe/resistant disease. The expanded series of ligands will consist of carbohydrate ligands (including trimannoside ligands to the CD 206 receptor of alveolar macrophages) for which binding to immune cells present in the BALF is known or expected. Known antibody-based ligands were used as controls.

It is expected that the proposed approach (including the development of biochips based on a series of ligands) will make it possible to quantify macrophage subpopulations (M1, M2, and their subtypes) in patients. These “new” specific carbohydrate-based ligands will thus provide about 50 additional “coordinates” according to which BALF typing can be performed. As expected, this will provide a large additional array of data for analysis, compared to the work going on in the world. The dataset used by other researchers only for known “antibody” ligands is semi-quantitative and insufficient for the purposes of typing as yet unknown and uncomplicated sub-populations. The analysis of these data in combination with personalized information from patients’ medical records will be carried out using both traditional methods and machine learning methods.

The developed diagnostic method using a series of specific ligands (BAL typing) for the analysis of subpopulations of activated M1 and M2 macrophages and their subtypes opens the way for the development of a personalized treatment approach for bronchial asthma. In the future, it will be used to develop treatment tactics aimed at regulating the balance of M1 and M2 macrophage subpopulations and their subtypes, which determine the severity and outcome of the disease.

Further, with a series of developed ligands specifically binding to certain subtypes of macrophages at our disposal, delivery systems for anti-inflammatory drugs of both standard and natural drug candidates (based on derivatives of plant extracts—the coumarin family, etc.) will be developed, which, in our preliminary experiments and the literature, have shown a significant macrophage-associated response to inflammation in bronchial asthma. We plan to develop an inhalation form with increased bioavailability. To bring this technology into clinical practice, the first step is to create reliable diagnostic tools (including the development of biochips based on a series of ligands). This involves developing microarrays with specific carbohydrate-based ligands that can capture and characterize the binding profiles of patients’ macrophages. To analyze this complex data, we will use a robust machine learning algorithm. This algorithm will identify different subpopulations of macrophages associated with favorable and unfavorable disease outcomes. By doing this, we can accurately predict how the disease will progress and help us justify therapeutic strategies. In the second phase, we will focus on developing drugs to treat the disease. We will identify and optimize drug delivery systems that target specific subpopulations of macrophages involved in the disease. We will use the identified binding ligands to synthesize ligand–drug conjugates. Next, we will test these drugs in vitro and in vivo to ensure they are effective and safe. Targeting macrophages and eosinophils, key cells involved in inflammation in asthma, could potentially lead to more effective and safer medicines. The ability to modulate the immune response and reduce side effects makes this technology attractive for developing new treatments for asthma, potentially improving the quality of life of patients and reducing dependence on existing, often not sufficiently effective, therapeutic strategies.

## Figures and Tables

**Table 1:** Designations and physico-chemical parameters of IR and fluorescent markers used for macrophage typing. PBS (0.01 M, pH 7.4). T = 37 °C.

Type	Designation	Molar Ratio of Constituents	Hydrodynamic Diameter **, nm	ζ-Potential **, mV	K_dis_ (ConA–Ligand), M *	Percentage of Polymer Binding by Model Cells, %	
						CD206+ Macrophage ***	CD206–HEK293T
IR markers	IR1 (Gal_Cyc_-PEG_5000_)	1:1	85 ± 7	+3 ± 1	(1.0 ± 0.1) × 10^−4^	27 ± 4	23 ± 5
	IR2 (Man_Lin_-PEG_5000_)	1:1			(2 ± 1) × 10^−3^	19 ± 2	14 ± 3
	IR3 (triMan-PEG_5000_)	1:1	90 ± 10		(8 ± 1) × 10^−7^	82 ± 3	20 ± 4
Fluorescent markers	Man_Lin_-PEI-FITC	15:1:1	110 ± 20	+10 ± 2	(7 ± 2) × 10^−4^	25 ± 3	11 ± 2
	Man_Cyc_-PEI-FITC	18:1:1			(1.5 ± 0.2) × 10^−5^	70 ± 6	18 ± 3
	Gal_Lin_-PEI-FITC	16:1:1			(3 ± 1) × 10^−3^	17 ± 2	10 ± 2
	Gal_Cyc_-PEI-FITC	13:1:1			(4 ± 1) × 10^−5^	64 ± 3	12 ± 5
	triMan-PEI-FITC	10:1:1	130 ± 15		(2.5 ± 0.3) × 10^−7^	90 ± 5	15 ± 4

* ConA—a model for CD206 receptor; ** measured by DLS; *** Derived from model THP-1 human monocytes.

**Table 2:** Selectivity of ligand binding (FITC-positive cells, %) with different affinities for CD206+ macrophages (model cell line and from BALF) in comparison with CD206− HEK293T cells according to flow cytometry. T = 37 °C.

FITC-Positive Cells, %	Man_Lin_-PEI-FITC	Man_Cyc_-PEI-FITC	Gal_Lin_-PEI-FITC	Gal_Cyc_-PEI-FITC	triMan-PEI-FITC	Mannan-FITC	Selectivity Index triMan/Man_Lin_ or Manan/Gal_Lin_
CD206+ macrophage derived from THP-1	29 ± 3	55 ± 6	22 ± 2	40 ± 3	88 ± 5	80 ± 6	4
CD206+ macrophage isolated from BALF	13 ± 5	22 ± 9	25 ± 4	32 ± 7	45 ± 10	52 ± 5	4
CD206− HEK293T	11 ± 2	16 ± 3	11 ± 2	12 ± 5	14 ± 4	14 ± 4	1.2

## Data Availability

The data presented in this study are available in the main text and the Supplementary Material.

## Supplementary Materials

The following supporting information can be downloaded at https://www.mdpi.com/article/10.3390/polym16233427/s1, Figure S1. The number of amino groups per putrescine molecule is determined initially and after modifications by TNBS titration. Sodium borate buffer (50 mM, pH 9.2). T = 22 °C. Figure S2. (a) FTIR spectra of the initial substances and the target IR marker triMan-PEG. PBS (0.01 M, pH 7.4). T = 22 °C. (b) ^1^H NMR spectra of the initial substances and the target IR marker Man-PEG. D_2_O. T = 22 °C. Figure S3. Normalized on Amide 1 peak FTIR spectra of BALF samples (from a patient with purulent endobronchitis) during real-time incubation with marker IR1. The volume of the reaction mixture is 35 µL. The number of cells from BALF is about 2 × 10^5^. The concentration of the IR marker is 5 mg/mL by PEG (equivalent to 1 mM). PBS (0.01 M, pH 7.4). T = 37 °C. Figure S4. (a) FTIR microscopic maps of the peak intensities integral distribution in the IR spectra of BALF samples (diagnosed with purulent endobronchitis) incubated for 1 h with different IR markers (10 mg/mL). (b) FTIR microscopic maps of the peak intensities integral distribution in the IR spectra of BALF samples (diagnosed with purulent endobronchitis) incubated for 1 h with different IR markers (10 mg/mL) followed by mannan treatment (10 mg/mL) for 1 h. PBS (0.01 M, pH 7.4). T = 37 °C. Figure S5. (a) FTIR microscopic maps of the peak intensities integral distribution in the IR spectra of BALF samples (from a patient with bronchial asthma (with plastic bronchitis)) incubated for 1 h with different IR markers (10 mg/mL). (b) FTIR microscopic maps of the peak intensities integral distribution in the IR spectra of BALF samples (diagnosed with purulent endobronchitis) incubated for 1 h with different IR markers (10 mg/mL) followed by mannan treatment (10 mg/mL) for 1 h. PBS (0.01 M, pH 7.4). T = 37 °C. Figure S6. CLSM images of (a) THP-1-derived macrophage-like cells and (b) human dermal fibroblasts (HDF). Phagocytosis assay with PEI-triMan-FITC and PEI-Man-FITC after incubation for 40 min (green channel). Phase contrast microscopy, fluorescent microscopy, blue channel—nuclei stained with DAPI, red channel—CD206 receptors were stained with Alexa594. Scale bar, 100 μm.

## Abbreviations

BALF	bronchoalveolar lavage fluid
BALF	bronchoalveolar lavage fluid
COPD	chronic obstructive pulmonary disease
DLS	dynamic light scattering
DMSO	dimethylsulfoxide
FITC	fluorescein isothiocyanate
FTIR	Fourier transformed infrared
Gal	galactose
GC-MS	gas chromatography–mass spectrometry
IR	infrared
Man	mannose
NMR	nuclear magnetic resonance
PEI	polyethyleneimine
PEG	polyethyleneglycol
