# Supplementary material for: Polymeric Infrared and Fluorescent Probes to Assess Macrophage Diversity in Bronchoalveolar Lavage Fluid of Asthma and Other Pulmonary Disease Patients
Source: Polymers (Basel). 2024 Dec 5;16(23):3427. doi: 10.3390/polym16233427 (PMC11644818; doi:10.3390/polym16233427)
Supplement: Supplementary file 1 [file polymers-16-03427-s001.zip › polymers-3283642-supplementary.pdf]

Polymeric infrared and fluorescent probes to assess macrophage diversity in bronchoalveolar lavage fluid of asthma and other pulmonary disease patients

Igor D. Zlotnikov [1] and Elena V. Kudryashova [1,*]

1 Faculty of Chemistry, Lomonosov Moscow State University, Leninskie Gory, 1/3, 119991 Moscow, Russia; zlotnikovid@my.msu.ru, nbelog@mail.ru (N.G.B.)
*Correspondence: Helenakoudriachova@yandex.ru (E.V.K.)

Figure S1. The number of amino groups per putrescine molecule is initially and after modifications determined by TNBS titration. Sodium borate buffer (50 mM, pH 9.2). T = 22 °C.

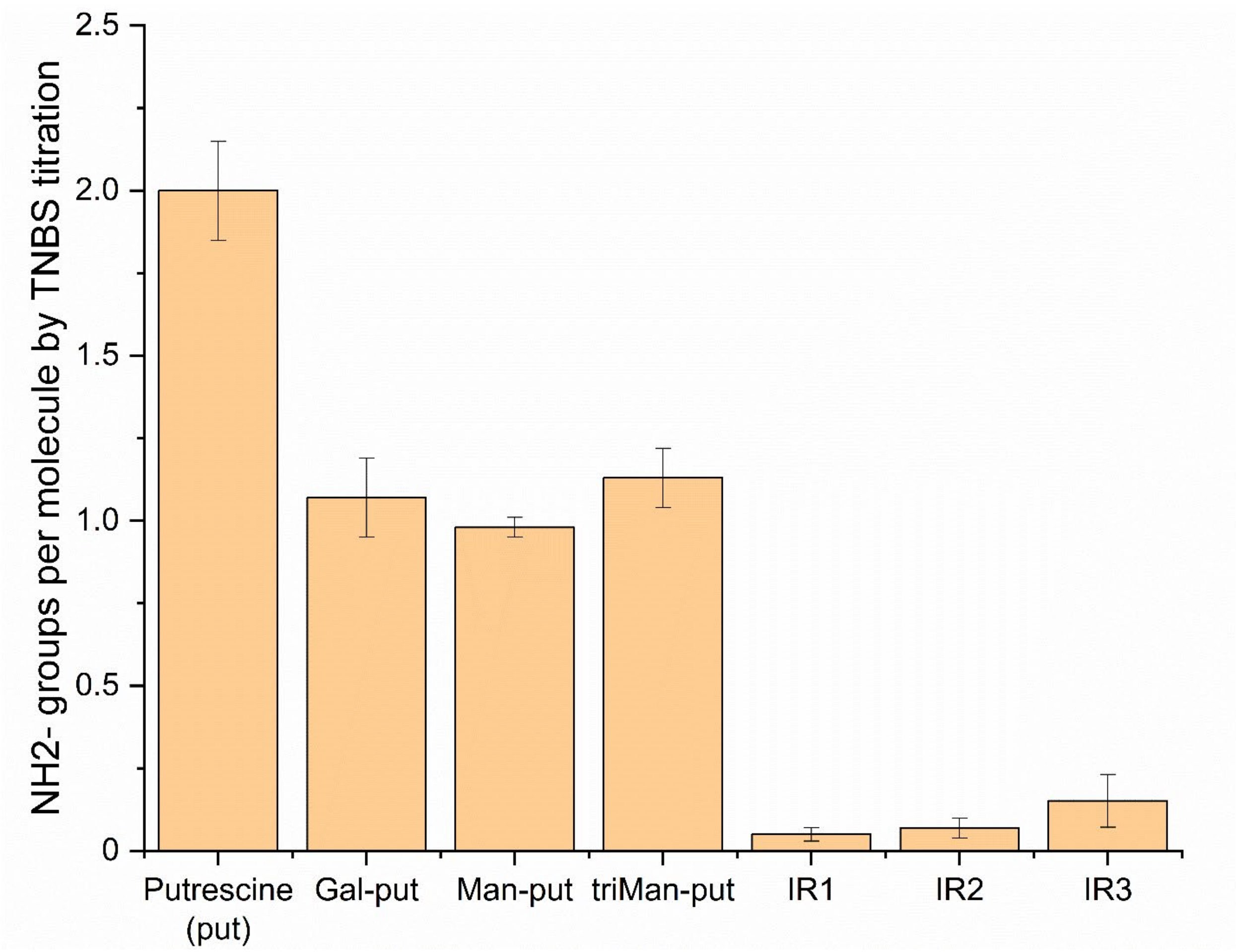

Figure S2. (a) FTIR spectra of the initial substances and the target IR marker triMan-PEG. PBS (0.01 M, pH 7.4). T = 22 °C. **(b)** ^{1}H NMR spectra of the initial substances and the target IR marker Man-PEG. D_2O. T = 22 °C.

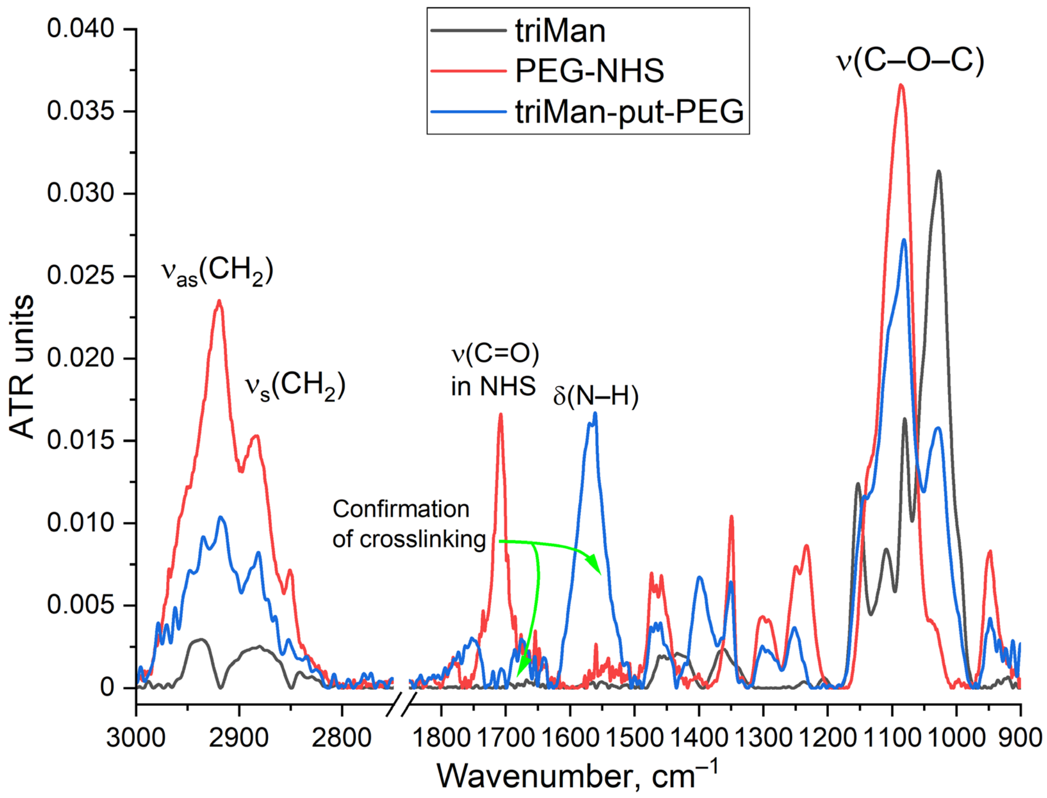

(a)

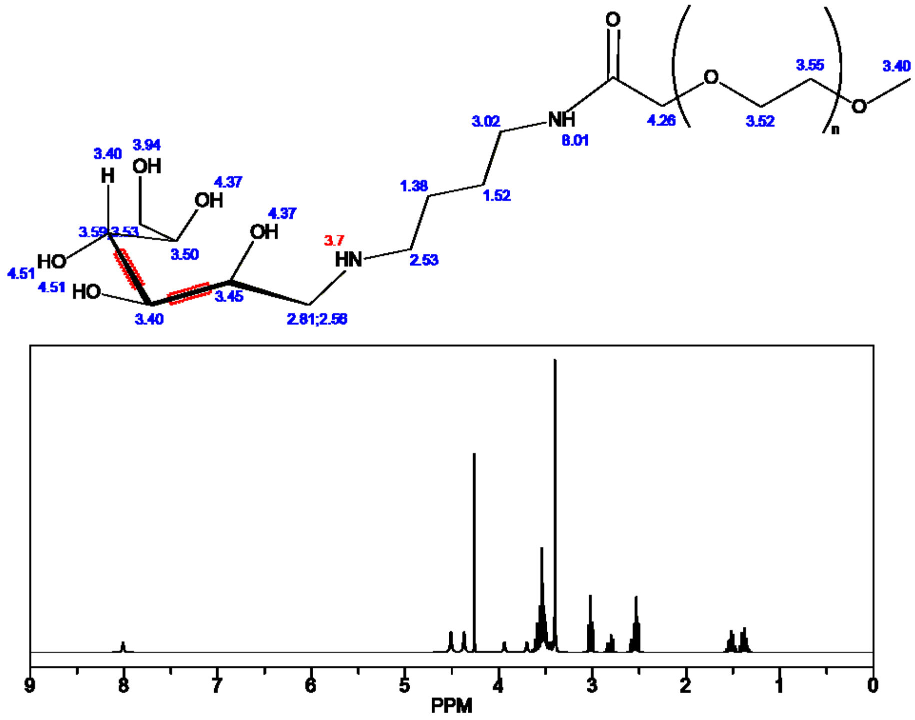

(b)

Figure S3. Normalized on Amide I peak FTIR spectra of BALF samples (from a patient with purulent endobronchitis) during real-time incubation with marker IR1. The volume of the reaction mixture is 35 µl. The number of cells from BALF is about 2×10[5]. The concentration of the IR marker is 5 mg/mL by PEG (equivalent to 1 mM). PBS (0.01M, pH 7.4). T = 37 °C.

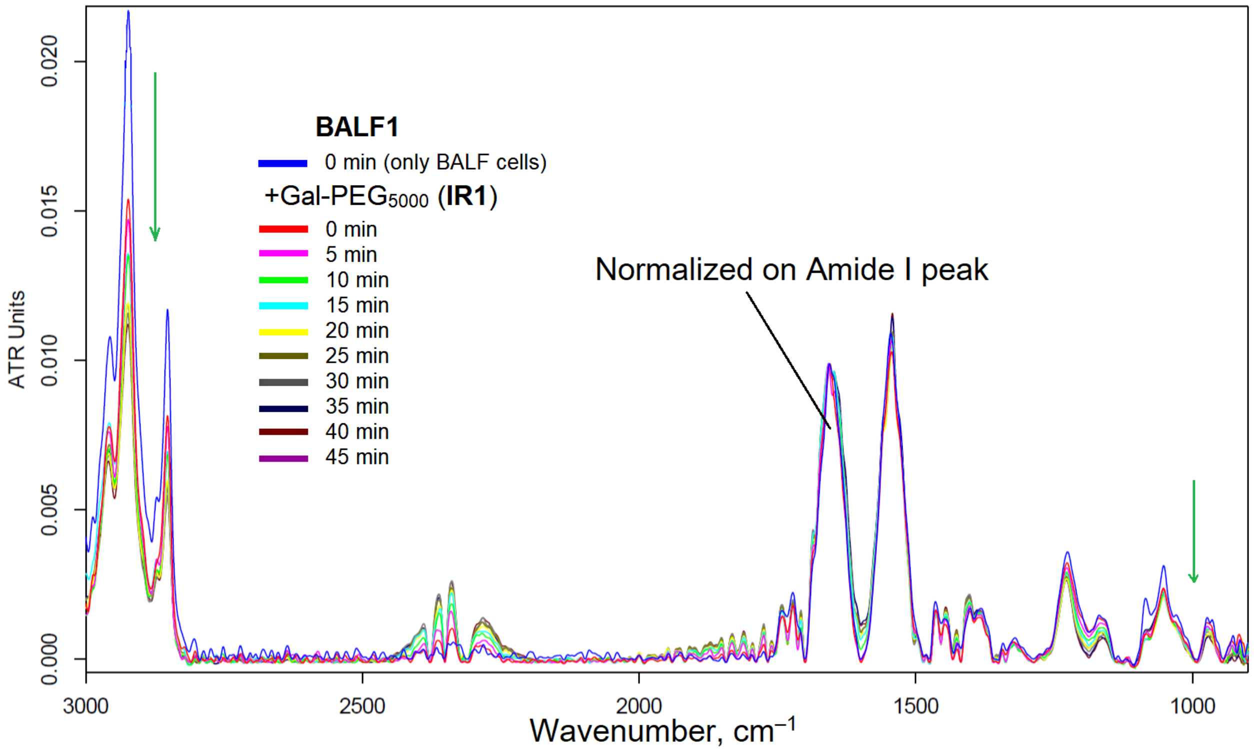

Figure S4. (a) FTIR microscopic maps of the peak intensities integral distribution in the IR spectra of BALF samples (diagnosed with purulent endobronchitis) incubated for 1h with different IR markers (10 mg/ml). **(b)** FTIR microscopic maps of the peak intensities integral distribution in the IR spectra of BALF samples (diagnosed with purulent endobronchitis) incubated for 1h with different IR markers (10 mg/ml) followed by mannan treatment (10 mg/ml) for 1 h. PBS (0.01M, pH 7.4). T = 37 °C.

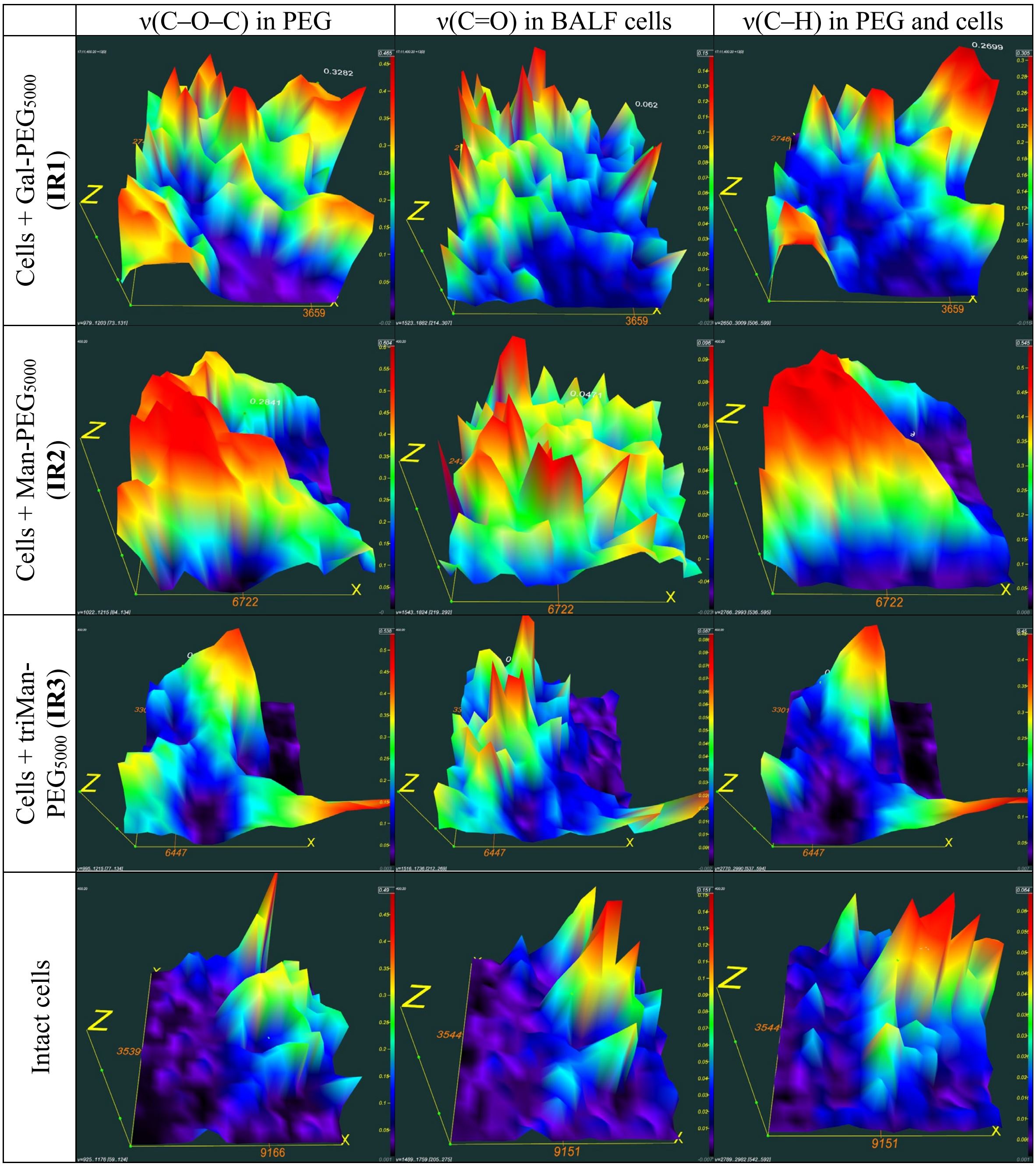

(a)

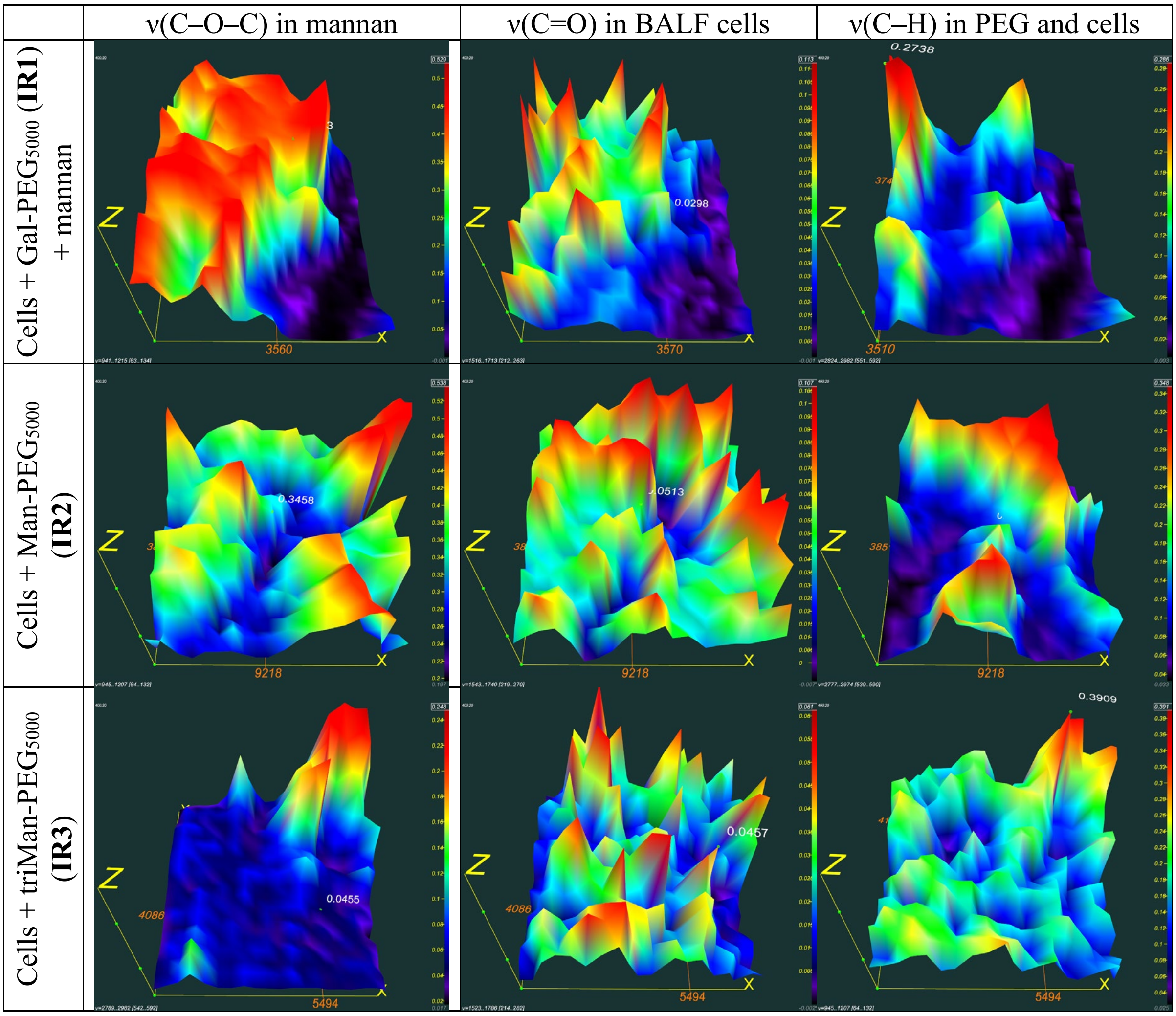
ν(C–O–C) in mannan
ν(C=O) in BALF cells
ν(C–H) in PEG and cells
Cells + Gal-PEG5000 (IR1) + mannan
Cells + Man-PEG5000 (IR2)
Cells + triMan-PEG5000 (IR3)
3560
3570
0.0298
0.2738
3510
9218
0.3458
9218
9218
0.0455
4086
5494
0.0457
4086
5494
0.3909
5494

(b)

Figure S5. (a) FTIR microscopic maps of the peak intensities integral distribution in the IR spectra of BALF samples (from a patient with bronchial asthma (with plastic bronchitis)) incubated for 1h with different IR markers (10 mg/ml). **(b)** FTIR microscopic maps of the peak intensities integral distribution in the IR spectra of BALF samples (diagnosed with purulent endobronchitis) incubated for 1h with different IR markers (10 mg/ml) followed by mannan treatment (10 mg/ml) for 1 h. PBS (0.01M, pH 7.4). T = 37 °C.

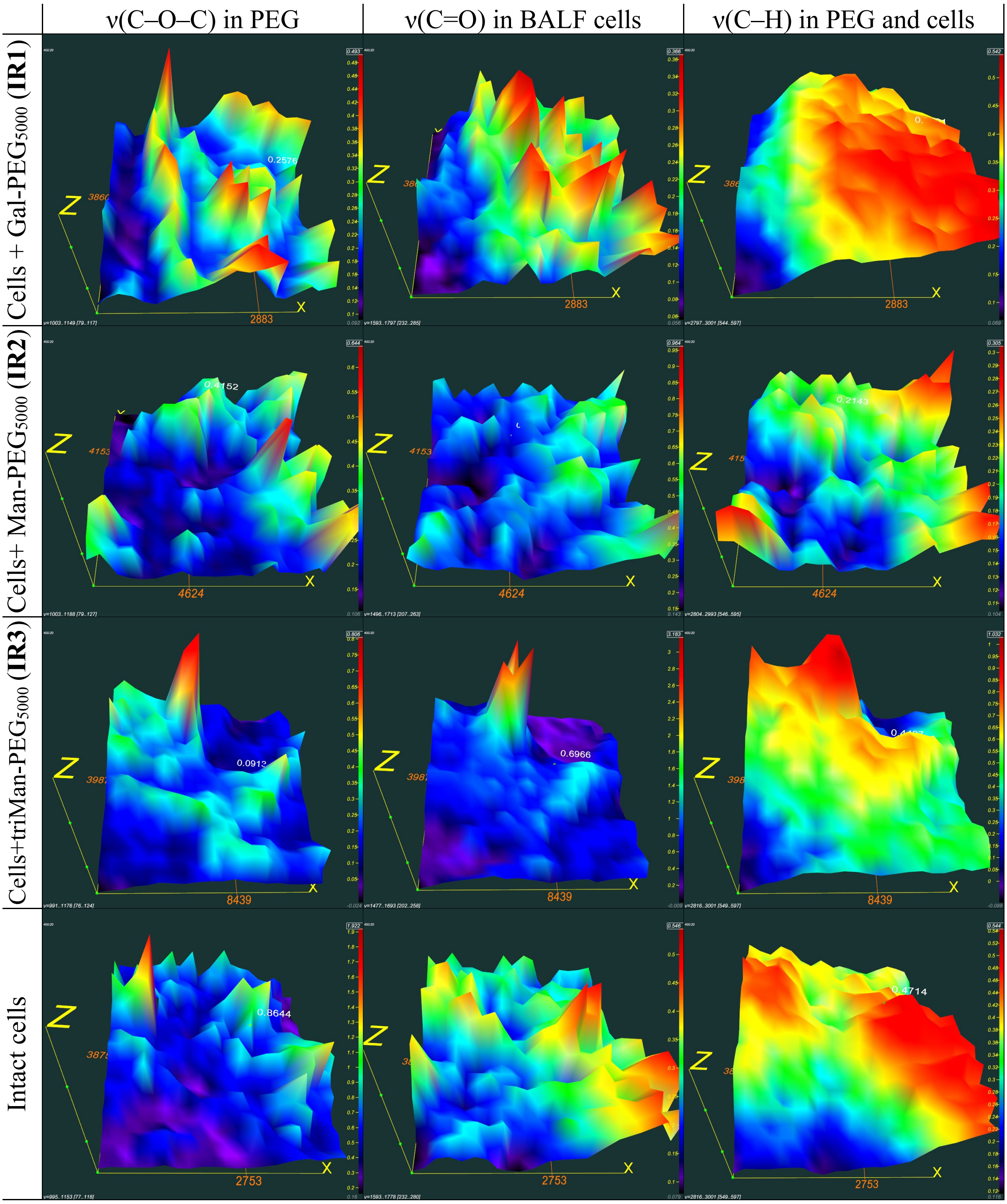

(a)

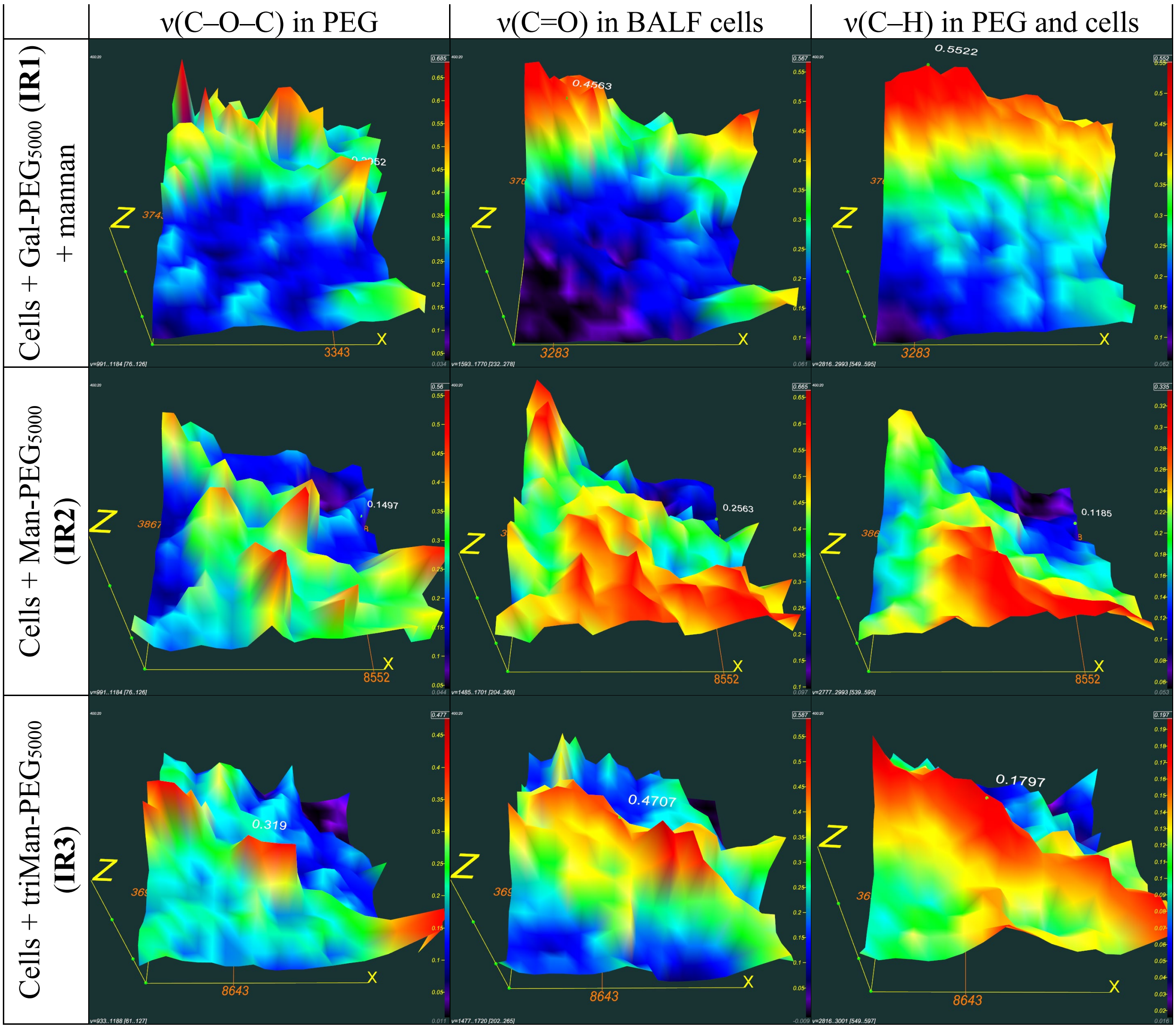
ν(C–O–C) in PEG
ν(C=O) in BALF cells
ν(C–H) in PEG and cells
Cells + Gal-PEG5000 (IR1) + mannan
Cells + Man-PEG5000 (IR2)
Cells + triMan-PEG5000 (IR3)
0.4563
0.5522
3343
3283
3283
0.1497
0.2563
0.1185
8552
8552
8552
0.319
0.4707
0.1797
8643
8643
8643

(b)

Figure S6. CLSM images of (a) THP-1-derived macrophage-like cells and (b) human dermal fibroblasts (HDF). Phagocytosis assay with PEI-triMan-FITC and PEI-Man-FITC after incubation for 40 min (green channel). Phase contrast microscopy, fluorescent microscopy, blue channel—nuclei stained with DAPI, red channel – CD206 receptors were stained with Alexa594. Scale bar, 100 μm.

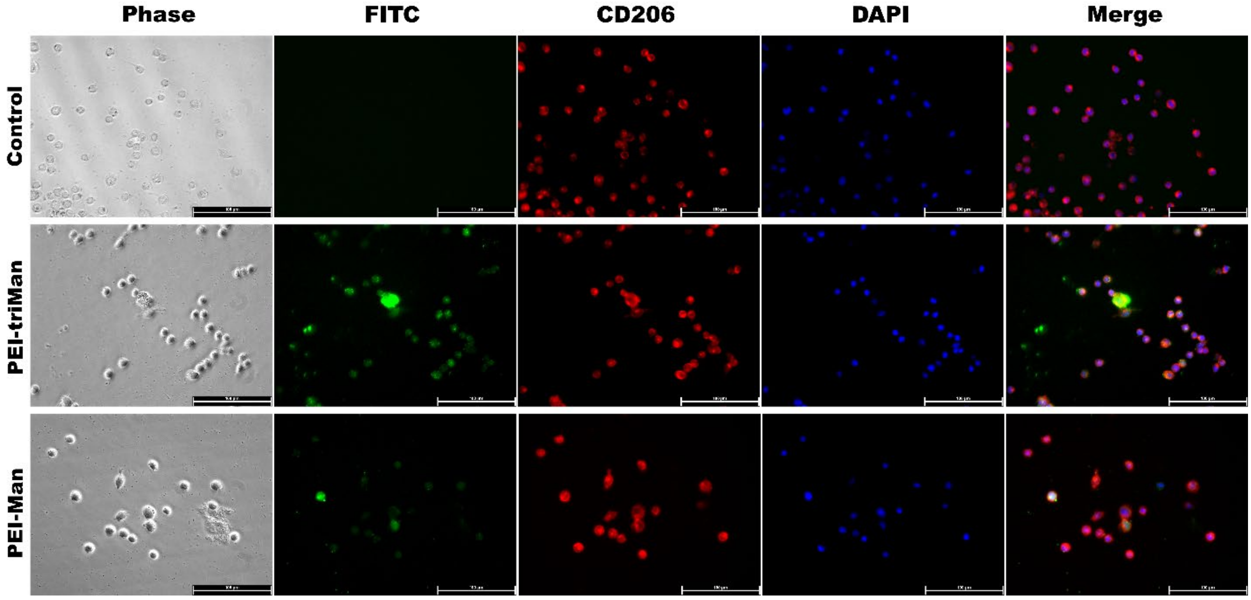

(a)

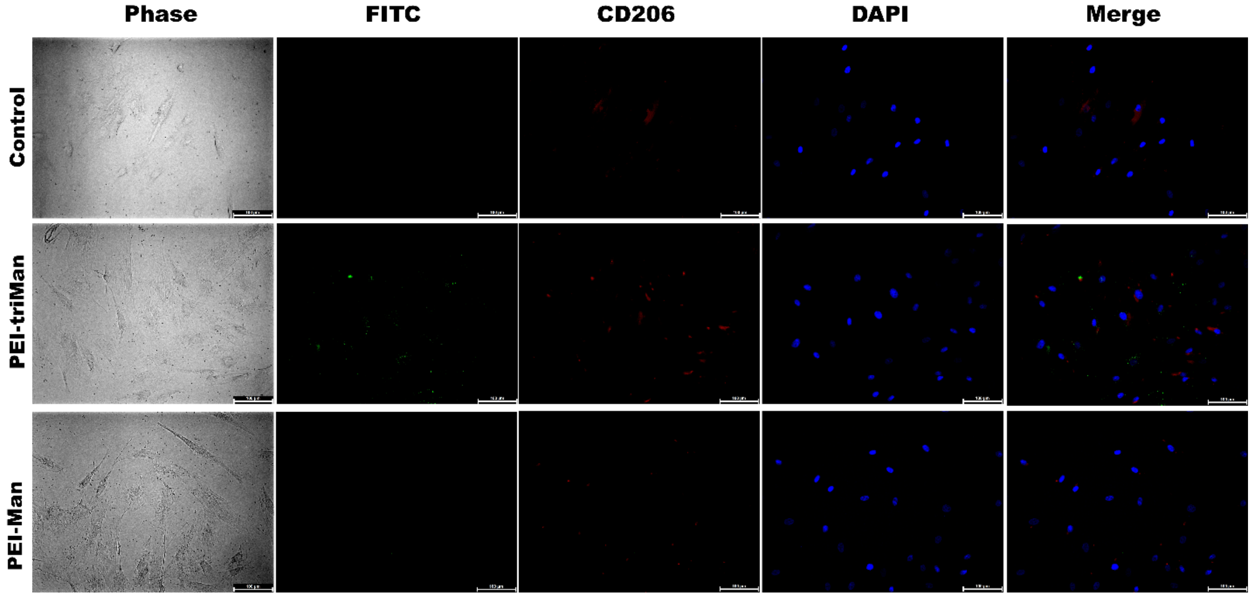

(b)
